# Inhibition of Non-flux-Controlling Enzymes Deters Cancer Glycolysis by Accumulation of Regulatory Metabolites of Controlling Steps

**DOI:** 10.3389/fphys.2016.00412

**Published:** 2016-09-23

**Authors:** Álvaro Marín-Hernández, José S. Rodríguez-Zavala, Isis Del Mazo-Monsalvo, Sara Rodríguez-Enríquez, Rafael Moreno-Sánchez, Emma Saavedra

**Affiliations:** Departamento de Bioquímica, Instituto Nacional de CardiologíaMexico City, Mexico

**Keywords:** cancer glycolysis, metabolic regulation, uncompetitive inhibition, feed-back inhibition, enolase inhibition, pyruvate kinase inhibition, oxamate

## Abstract

Glycolysis provides precursors for the synthesis of macromolecules and may contribute to the ATP supply required for the constant and accelerated cellular duplication in cancer cells. In consequence, inhibition of glycolysis has been reiteratively considered as an anti-cancer therapeutic option. In previous studies, kinetic modeling of glycolysis in cancer cells allowed the identification of the main steps that control the glycolytic flux: glucose transporter, hexokinase (HK), hexose phosphate isomerase (HPI), and glycogen degradation in human cervix HeLa cancer cells and rat AS-30D ascites hepatocarcinoma. It was also previously experimentally determined that simultaneous inhibition of the non-controlling enzymes lactate dehydrogenase (LDH), pyruvate kinase (PYK), and enolase (ENO) brings about significant decrease in the glycolytic flux of cancer cells and accumulation of intermediate metabolites, mainly fructose-1,6-bisphosphate (Fru1,6BP), and dihydroxyacetone phosphate (DHAP), which are inhibitors of HK and HPI, respectively. Here it was found by kinetic modeling that inhibition of cancer glycolysis can be attained by blocking downstream non flux-controlling steps as long as Fru1,6BP and DHAP, regulatory metabolites of flux-controlling enzymes, are accumulated. Furthermore, experimental results and further modeling showed that oxamate and iodoacetate inhibitions of PYK, ENO, and glyceraldehyde3-phosphate dehydrogenase (GAPDH), but not of LDH and phosphoglycerate kinase, induced accumulation of Fru1,6BP and DHAP in AS-30D hepatoma cells. Indeed, PYK, ENO, and GAPDH exerted the highest control on the Fru1,6BP and DHAP concentrations. The high levels of these metabolites inhibited HK and HPI and led to glycolytic flux inhibition, ATP diminution, and accumulation of toxic methylglyoxal. Hence, the anticancer effects of downstream glycolytic inhibitors are very likely mediated by this mechanism. In parallel, it was also found that uncompetitive inhibition of the flux-controlling steps is a more potent mechanism than competitive and mixed-type inhibition to efficiently perturb cancer glycolysis.

## Introduction

In recent years it has been extensively documented that oxidative phosphorylation (OxPhos) is predominant for supplying ATP in cancer cells under aerobic conditions (Zu and Guppy, 2004; Moreno-Sánchez et al., 2007; Ralph et al., 2010). However, cancer glycolysis becomes prevalent when OxPhos is down-regulated by long-term hypoxia or high incidence of mutations in mitochondrial DNA (Carew and Huang, 2002; Gatenby and Gillies, 2004; Rodríguez-Enríquez et al., 2010; Hernández-Reséndiz et al., 2015). Glycolysis also provides precursors for the synthesis of the macromolecules required for the constant and accelerated cellular duplication of cancer cells (Bauer et al., 2005). In addition, the enhanced lactic acid (a glycolytic end-product) production and secretion by cancer cells has been proposed to promote evasion of the immune system and induction of angiogenesis and metastasis (Lardner, 2001; Fischer et al., 2007). In consequence, glycolysis inhibition has re-emerged as an alternative therapeutic option for cancer (Warmoes and Locasale, 2014). In addition, cancer cells may induce oxidative stress on neighboring stromal fibroblasts triggering mitophagy and hence re-directing their energy metabolism toward glycolysis. In return, the lactate produced and expelled by fibroblasts, as well as ketone bodies, are now taken up and actively oxidized by cancer cells to drive OxPhos, which presumably favors tumor growth. This cell-cell interplay has been called reverse Warburg effect (Pavlides et al., 2009; Martinez-Outschoorn et al., 2011).

By applying the fundamentals of metabolic control analysis (Fell, 1997; Moreno-Sánchez et al., 2008, 2010), the enzymes and transporters that control the glycolytic flux of cancer cells have been identified. These are indeed the targets with the highest therapeutic potential because their inhibition will have greater negative effects on tumor glycolysis than inhibition of low- or negligible flux-controlling steps. It was determined by both, elasticity analysis and kinetic modeling (experimental strategies of metabolic control analysis and bottom-up Systems Biology, respectively), that the main controlling steps of cancer glycolysis are the glucose transporter (GLUT), hexokinase (HK), hexose phosphate isomerase (HPI), and glycogen degradation, regardless the environmental conditions to which the cells were exposed (normoxia/normoglycemia, hypoxia/hyperglycemia, and normoxia/hypoglycemia; Marín-Hernández et al., 2006, 2011, 2014). Although the degree of flux control exerted by these controlling steps slightly changes among the different conditions, the main controlling steps remain the same, which emphasizes the fact that cancer glycolysis is also tightly regulated despite its flux enhancement.

However, non-flux-controlling glycolytic steps such as glyceraldehyde-3-phosphate dehydrogenase (GAPDH), pyruvate kinase (PYK), and lactate dehydrogenase (LDH) have been also proposed as suitable targets for inhibition of cancer glycolysis (Ganapathy-Kanniappan et al., 2012; Ganapathy-Kanniappan and Geschwind, 2013; Daniele et al., 2015). Inhibition of any of these three non-controlling enzymes induces a moderate decrease in the growth of cancer cells (Tang et al., 2012; Daniele et al., 2015). However, this anticancer effect could be rather linked to inhibition of the “moonlighthing” or accessory functions of glycolytic enzymes which include roles in cancer development and promotion and cell cycle progression (Ganapathy-Kanniappan and Geschwind, 2013; Hu et al., 2014).

On the other hand, the glycolytic metabolites glucose-6-phosphate (Glc6P) and fructose-1,6-bisphosphate (Fru1,6BP) and the pentose phosphate pathway metabolites erythrose-4-phosphate (Ery4P) and 6-phosphogluconate (6PG) can modulate the activities of the controlling enzymes HK and HPI through competitive and mixed-type inhibitions. Furthermore, some metabolites that at low, physiological concentrations are innocuous, at high concentrations may become inhibitors of the controlling steps HK (Fru1,6BP) and HPI (dihydroxyacetone-phosphate; DHAP), inducing significant inhibition of the glycolytic flux in cancer cells (Moreno-Sánchez et al., 2016). Elevated levels of Fru1,6BP and DHAP in cancer cells can be achieved by inhibiting, simultaneously, down-stream enzymes with negligible flux-control such as enolase (ENO), PYK, and LDH. Therefore, inhibitors of these latter enzymes may also function as anti-glycolytic drugs because they may indirectly induce inhibition of the high flux-controlling HK and HPI. To understand the mechanistic basis of why inhibition of down-stream non-controlling glycolytic enzymes may affect the pathway flux, it appears necessary to determine which are the down-stream steps with high control on the concentrations of the regulatory metabolites Fru1,6BP and DHAP (i.e., metabolite concentration control coefficients).

Such a goal was pursued and resolved in the present paper by using our published AS-30D and HeLa cells glycolysis kinetic models (Marín-Hernández et al., 2011, 2014; Moreno-Sánchez et al., 2016). Previous theoretical studies have suggested that uncompetitive inhibition induces more severe toxic effects on a metabolic pathway than competitive inhibition (Cornish-Bowden, 1986; Eisenthal and Cornish-Bowden, 1998). Therefore, *in silico* simulations of how different mechanisms of inhibition (competitive, mixed-type, uncompetitive) on controlling enzymes impact the pathway systemic properties (fluxes and metabolite concentrations) were also carried out using the kinetic glycolysis models.

It was concluded that (i) inhibition of GAPDH with iodoacetate, or PYK/ENO with oxamate but not LDH, PGK, or PGAM, induces Fru1,6BP and DHAP accumulation and methylglyoxal production, leading to significant suppression of glycolysis; and (ii) uncompetitive inhibition of the most controlling pathway steps is the most direct and potent mechanism to efficiently perturb cancer glycolysis.

## Materials and methods

### Chemicals

HK, Glc6PDH, HPI, aldolase, α-glycerophosphate dehydrogenase, triosephosphate isomerase (TPI), LDH, and Fru6P were purchased from Roche (Mannheim, Germany). Glucose, iodoacetate, methylglyoxal, NADH, NAD^+^, NADP^+^, and oxamate were from Sigma Chemical (St Louis, MO, USA).

### Isolation of tumor cells

AS-30D hepatocarcinoma cells were propagated in 200–250 g weight female Wistar rats by intraperitoneal inoculation of 3 mL of ascitic liquid containing ~4–6 × 10^8^ cells/mL. After 5–6 days, the intraperitoneal cavity liquid was extracted and tumor cells were isolated by centrifugation as previously described (López-Gómez et al., 1993). Animal manipulation was carried out in accordance with the recommendations of Mexican Official Standard NOM-062-ZOO-1999. This study did not require approval by the Ethics Committee of the Instituto Nacional de Cardiología de México.

### Glycolytic fluxes and metabolite concentrations

Hepatocarcinoma AS-30D cells (15 mg cell protein/mL) were incubated in saline Krebs-Ringer medium supplied with oxamate (10 or 20 mM) or iodoacetate (2 or 4 mM) for 60 min under orbital shaking at 150 rpm and 37°C; under such conditions cell viability was always higher than 90%. Thereafter, a cell sample was withdrawn (time 0) and 5 mM glucose was added; after further 10 min of incubation another cell sample (time 10) was withdrawn. The cell samples were immediately mixed with ice-cold perchloric acid (final concentration of 3% v/v), vortexed and centrifuged at 1800 × g for 1 min at 4°C. The supernatants were neutralized with 3 M KOH/0.1 M Tris, further incubated in ice for at least 30 min and then centrifuged. The supernatants were stored at −72°C until use for determination of Glc6P, Fru6P, Fru1,6BP, G3P, DHAP, ATP, ADP, and L-lactate contents as described by Bergmeyer (1974). The rate of the glycolytic flux was estimated from the difference in L-lactate contents from the *t* = 0 and *t* = 10 min samples. As glycogen degradation and glutaminolysis are negligible in AS-30D cells (Marín-Hernández et al., 2006), total L-lactate production did not require correction provided by 2-DOG inhibition.

Methylglyoxal was determined by gas chromatography in a Shimadzu GC2010 apparatus (Shimadzu; Kyoto, Japan) equipped with a capillary column HP-PLOT/U of 30 m length, 0.32 mm I.D. and 10 μm film (Agilent, USA), and flame ionization detector. A methylglyoxal standard curve was carried out in the range of 0.3–30 nmoles, and the time of retention was 4.7 min. The equipment conditions were FID temperature 200°C, column temperature 180°C, oven temperature 180°C, and linear velocity 26.4 cm/s. He (10 ml/min) and H_2_ (40 ml/min) mix was used as carrier gas. The cell sample (15 mg/ml) was withdrawn after time 10 and centrifuged at 1800 × g for 2 min. 0.5 mL of the supernatant was removed and the cell pellet was resuspended in the remaining supernatant. The suspension was sonicated with a Branson sonicator three times for 15 s at 60% of maximal output with 1 min rest, in an ice bath. The sonicate was centrifuged at 20 800 × g for 5 min. The supernantant was filtered and 1–2 μl were injected in the gas chromatograph. The limit of detection of methylglyoxal was lower than 0.3 nmoles. The concentration in the stock solution of methlyglyoxal was enzymatically calibrated by using human ALDH2 and saturating NAD^+^.

### Kinetic modeling

The previous kinetic models of glycolysis built for HeLa and AS-30D cells (Marín-Hernández et al., 2014; Moreno-Sánchez et al., 2016) using the metabolic simulator GEPASI version 3.3 (Mendes, 1993) were modified for the HK, HPI, TPI, and GAPDH rate equations as described below. The other rate equations remained unaltered, however, they are here fully described (Supplementary Table 1) because model updates previously developed are scattered in several papers (Marín-Hernández et al., 2011, 2014; Moreno-Sánchez et al., 2016). The models and simulations were also run in COPASI software (Hoops et al., 2006) with no significant differences to those of GEPASI (SBML files are included in Supplementary Presentation 1). The great majority of the kinetic parameter values used in the models were determined under the same experimental conditions (K^+^-based medium at pH 7.0 and 37°C; Marín-Hernández et al., 2006, 2011, 2014; Rodríguez-Enríquez et al., 2009; Moreno-Sánchez et al., 2012, 2016).

In the AS-30D model, kinetics of GLUT was defined as a monosubstrate reversible Michaelis-Menten equation [Haldane equation (Equation 1)] as it was previously determined (Rodríguez-Enríquez et al., 2009; Marín-Hernández et al., 2011):

(1) $$v=\frac{Vmf\left(\left[Glc_{out}\right]-\frac{\left[Glc_{in}\right]}{Keq}\right)}{K_{Glcout}\left(1+\frac{\left[Glc_{in}\right]}{K_{Glcin}}\right)+Glc_{out}}$$

in which *Glc*_*out*_ and *Glc*_*in*_ and *K*_*Glcout*_ and *K*_*Glcin*_ are the extra- and intra-cellular glucose concentrations and the *Km* values, respectively; *Keq* is the equilibrium constant; and *Vmf* is the maximal velocity in the forward reaction.

In the HeLa model, the rate-equation for GLUT was changed to a double mono-substrate reversible Michaelis-Menten equation (Equation 2), representing the co-existence of two isoforms (Marín-Hernández et al., 2014),

(2) $$\begin{array}{l} v=Vmf(\left[\frac{f1\left(\left[Glc_{out}\right]-\frac{\left[Glc_{in}\right]}{Keq}\right)}{K_{Glcout1}\left(1\;+\frac{\left[Glc_{in}\right]}{K_{Glcin1}}\right)\;+\;Glc_{out}}\right]\\ \;+\;\left[\frac{f2\left(\left[Glc_{out}\right]-\frac{\left[Glc_{in}\right]}{Keq}\right)}{K_{Glcout2}\left(1\;+\;\frac{\left[Glc_{in}\right]}{K_{Glcin2}}\right)\;+\;Glc_{out}}\right]) \end{array}$$

in which *K*_*Glcout*1_ and *K*_*Glcout*2_ are the *Km* values for extracellular glucose of each GLUT isoform; and *K*_*Glcin*1_ and *K*_*Glcin*2_ are the *Km* values for intracellular glucose of each GLUT isoform. *f* 1 and *f* 2 are the fractional isoform contents determined by Western blot analysis and enzyme kinetics (Marín-Hernández et al., 2014). This two-components equation was proposed in the previous study because cells grown in low glucose express significant contents of both isoforms, GLUT1 and GLUT3 (Marín-Hernández et al., 2014).

The HK rate equation (Equation 3) used for the present updated AS-30D model was a random Bi-Bi system (Segel, 1975) with mixed type inhibition by Fru1,6BP based on previous experimental kinetic analysis (Moreno-Sánchez et al., 2016):

(3) $$v=\frac{\frac{Vmf}{\alpha 1KaKb}\left(\left[A\right]\left[B\right]-\frac{\left[P\right]\left[Q\right]}{Keq}\right)}{1\;+\;\frac{\left[A\right]}{Ka}\;+\;\frac{\left[B\right]}{Kb}\;+\;\frac{\left[A\right]\left[B\right]}{\alpha 1KaKb}\;+\;\frac{\left[P\right]}{Kp}\;+\;\frac{\left[Q\right]}{Kq}\;+\;\frac{\left[P\right]\left[Q\right]}{KpKq}\;+\;\frac{\left[A\right]\left[Q\right]}{KaKq}\;+\;\frac{\left[P\right]\left[B\right]}{KpKb}\;+\;\frac{\left[I\right]}{Ki}\;+\;\frac{\left[A\right]\left[I\right]}{\alpha_{2}KaKi}\;+\;\frac{\left[A\right]\left[I\right]\left[B\right]}{\alpha 1KaKb\alpha_{2}Ki}}$$

where *A* = [Glc_in_], *B* = [ATP], *P* = [Glc6P] and *Q* = [ADP]. *Ka, Kb, Kp*, and *Kq* are the *Km* values for the corresponding substrates and products. α_1_ and α_2_ values are the factors by which *Ka* (*Km*_glc_) changes when *B* (ATP) and *I* (Fru1,6BP) are bound to the enzyme, respectively. *Ki* is the inhibition constant for Fru1,6BP (*Ki*_Fru1, 6BP_).

In the HeLa model, the HK rate equation (Equation 4) was a double random-bisubstrate Michaelis-Menten to also represent the coexistence of two enzyme isoforms as previously reported (Marín-Hernández et al., 2014),

(4) $$\begin{array}{l} v=Vmf(\left[\frac{\frac{f1}{Ka1Kb}\left(\left[A\right]\left[B\right]\;-\;\frac{\left[P\right]\left[Q\right]}{Keq}\right)}{1\;+\;\frac{\left[A\right]}{Ka1}\;+\;\frac{\left[B\right]}{Kb}\;+\;\frac{\left[A\right]\left[B\right]}{Ka1Kb}\;+\;\frac{\left[P\right]}{Kp}\;+\;\frac{\left[Q\right]}{Kq}\;+\;\frac{\left[P\right]\left[Q\right]}{KpKq}\;+\;\frac{\left[A\right]\left[Q\right]}{Ka1Kq}\;+\;\frac{\left[P\right]\left[B\right]}{KpKb}}\right]\\ \;+\left[\frac{\frac{f2}{Ka2Kb}\left(\left[A\right]\left[B\right]-\frac{\left[P\right]\left[Q\right]}{Keq}\right)}{1\;+\;\frac{\left[A\right]}{Ka2}\;+\;\frac{\left[B\right]}{Kb}\;+\;\frac{\left[A\right]\left[B\right]}{Ka2Kb}\;+\;\frac{\left[P\right]}{Kp}\;+\;\frac{\left[Q\right]}{Kq}\;+\;\frac{\left[P\right]\left[Q\right]}{KpKq}\;+\;\frac{\left[A\right]\left[Q\right]}{Ka2Kq}\;+\;\frac{\left[P\right]\left[B\right]}{KpKb}}\right]) \end{array}$$

in which *Ka1* and *Ka2* represent the *Km* values for Glc_in_ of each isoform; *f* 1 and *f* 2 are the fractional isoform contents experimentally determined from the activities of HKI and HKII in HeLa cellular extracts (Marín-Hernández et al., 2014).

The HPI rate equation in the HeLa model was considered as a monoreactant reversible Michaelis-Menten equation (Equation 5) with (a) competitive (Marín-Hernández et al., 2011, 2014), (b) uncompetitive (Segel, 1975), and (c) mixed type inhibition (Segel, 1975) by Ery4P, 6PG, and Fru1,6BP.

(5) $$\begin{array}{l} (\text{a})\;v=\frac{V_{mf}\frac{\left[Glc6P\right]}{K_{glc6p}}\;-\;V_{mr}\frac{\left[Fru6P\right]}{K_{fru6p}}}{1\;+\;\frac{\left[Glc6P\right]}{K_{glc6p}}\;+\;\frac{\left[Fru6P\right]}{K_{fru6p}}\;+\;\frac{\left[ERY4P\right]}{K_{ery4p}}\;+\;\frac{\left[Fru1,6BP\right]}{K_{fru1,6bp}}\;+\;\frac{\left[6PG\right]}{K_{6pg}}}\\ (\text{b})\;v=\frac{\frac{Vmf}{K_{glc6p}}\left[Glc6P\right]-\frac{Vmr}{K_{fru6p}}\left[Fru6P\right]}{\;1\;+\;\left(\frac{\left[Glc6P\right]}{K_{glc6p}}\right)*\left(1\;+\;\frac{\left[ERY4P\right]}{K_{ery4p}}\;+\;\frac{\left[Fru1,6BP\right]}{K_{fru1,6bp}}\text{ + }\frac{\left[6PG\right]}{K_{6pg}}\right)\;+\;\left(\frac{\left[Fru6P\right]}{K_{fru6p}}\right)*\left(1\;+\;\frac{\left[ERY4P\right]}{K_{ery4p}}+\;\frac{\left[Fru1,6BP\right]}{K_{fru1,6bp}}\;+\;\frac{\left[6PG\right]}{K_{6pg}}\right)}\\ (\text{c})\;v=\frac{Vmf=\frac{\left[Glc6P\right]}{K_{glc6p}\left(1\;+\;\frac{\left[ERY4P\right]}{K_{ery4p}}\;+\;\frac{\left[Fru1,6BP\right]}{K_{fru1,6bp}}\;+\;\frac{\left[6PG\right]}{K_{6pg}}\right)}-Vmr\frac{\left[Fru6P\right]}{K_{fru6p}\left(1\;+\;\frac{\left[ERY4P\right]}{K_{ery4p}}\;+\;\frac{\left[Fru1,6BP\right]}{K_{fru1,6bp}}\;+\;\frac{\left[6PG\right]}{K_{6pg}}\right)}}{1\;+\;\left(\frac{\left[Glc6P\right]}{K_{glc6p}}\right)*\left(\frac{1\;+\;\frac{\left[ERY4P\right]}{\propto K_{ery4p}}\;+\;\frac{\left[Fru1,6BP\right]}{\propto K_{fru1,6bp}}\;+\;\frac{\left[6PG\right]}{\propto K_{6pg}}}{1\;+\;\frac{\left[ERY4P\right]}{K_{ery4p}}\;+\;\frac{\left[Fru1,6BP\right]}{K_{fru1,6bp}}\;+\;\frac{\left[6PG\right]}{K_{6pg}}}\right)\;+\;\left(\frac{\left[Fru6P\right]}{K_{fru6p}}\right)*\left(\frac{1\;+\;\frac{\left[ERY4P\right]}{\propto K_{ery4p}}\;+\;\frac{\left[Fru1,6BP\right]}{\propto K_{fru1,6bp}}\;+\;\frac{\left[6PG\right]}{\propto K_{6pg}}}{1\;+\;\frac{\left[ERY4P\right]}{K_{ery4p}}\;+\;\frac{\left[Fru1,6BP\right]}{K_{fru1,6bp}}\;+\;\frac{\left[6PG\right]}{K_{6pg}}}\right)} \end{array}$$

The HPI rate equation in the AS-30D model was a monoreactant reversible equation (Equation 6) with competitive inhibition by four modulators: Ery4P, 6PG, Fru1,6BP and DHAP as experimentally determined (Moreno-Sánchez et al., 2016):

(6) $$v=\frac{Vmf\frac{\left[Glc6P\right]}{K_{glc6p}}\;-\;Vmr\frac{\left[Fru6P\right]}{K_{fru6P}}}{1\text{ ​}+\text{ ​}\frac{\left[Glc6P\right]}{K_{glc6p}}\text{ ​}+\text{ ​}\frac{\left[Fru6P\right]}{K_{fru6p}}\text{​ }+\text{ ​}\frac{\left[ERY4P\right]}{K_{ery4p}}\text{​ }+\text{ ​}\frac{\left[Fru1,6BP\right]}{K_{fru1,6bp}}\text{ ​}+\text{ ​}\frac{\left[PG\right]}{K_{6pg}}\text{​ }+\text{ ​}\frac{\left[DHAP\right]}{K_{dhap}}}$$

The rate equation for PFK-I (Equation 7) in all kinetic models was the concerted transition model of Monod, Wyman and Changeux for exclusive ligand binding (Fru6P, activators, and inhibitors) together with mixed-type activation and simple Michaelis–Menten terms for ATP and reverse reaction (Marín-Hernández et al., 2011, 2014) as established by experimental kinetic analysis (Moreno-Sánchez et al., 2012). ATP and citrate are the allosteric inhibitors. L is the allosteric transition constant; *Ka*_Fru26BP_ is the activation constant for Fru26BP; *Ki*_CIT_ and *Ki*_ATP_ are the inhibition constants for citrate and ATP; α and β are the factors by which *K*_Fru6P_ and *Vmax* change when a mixed-type activator is bound to the active enzyme.

Probably derived from the high complexity of the PFK1 rate equation, the algorithms used by COPASI to generate the ordinary differential equations to calculate the variation in the metabolite concentrations assign the role of the first substrate (or product) to that defined in the reaction specification window. If the order of substrates and products in the reaction does not match with that stated in the rate equation, then the computer program mix-up the identity of the ligands in the ordinary differential equations. Therefore, to correct for this type of errors both the syntax reaction and the rate equation have to be displayed in the same order of substrates and products; then one should be aware that the reaction syntax does not necessarily reflect the order of binding in the enzyme, which defines the type of reaction mechanism.

Reaction: ATP + Fru6P = ADP + Fru1,6BP

(7) $$\begin{array}{l} v=Vm(\left(\frac{\frac{\left[ATP\right]}{K_{ATP}}}{1\;+\;\frac{\left[ATP\right]}{K_{ATP}}}\right)\left(\frac{1\;+\;\frac{\beta Fru26BP}{\alpha Ka_{Fru26BP}}}{1\;+\;\frac{Fru26BP}{\alpha Ka_{Fru26BP}}}\right)\left(\frac{\frac{Fru6P\left(1\;+\;\frac{Fru26BP}{\alpha Ka_{Fru26BP}}\right)}{K_{Fru6P}\left(1\;+\;\frac{Fru26BP}{Ka_{Fru26BP}}\right)}{\left[1\;+\;\frac{Fru6P\left(1\;+\;\frac{Fru26BP}{\alpha Ka_{Fru26BP}}\right)}{K_{Fru6P}\left(1\;+\;\frac{Fru26BP}{Ka_{Fru26BP}}\right)}\right]}^{3}}{\frac{L{\left(1\;+\;\frac{\left[CIT\right]}{Ki_{CIT}}\right)}^{4}{\left(1\;+\;\frac{\left[ATP\right]}{Ki_{ATP}}\right)}^{4}}{{\left(1\;+\;\frac{Fru26BP}{Ka_{Fru26BP}}\right)}^{4}}\;+\;{\left[1\;+\;\frac{Fru6P\left(1\;+\;\frac{Fru26BP}{\alpha Ka_{Fru26BP}}\right)}{K_{Fru6P}\left(1\;+\;\frac{Fru26BP}{Ka_{Fru26BP}}\right)}\right]}^{4}}\right)\\ \;-\;\left(\frac{\frac{\left[ADP\right]\left[Fru16BP\right]}{K_{ADP}K_{Fru16BP}Keq}}{\frac{\left[ADP\right]}{K_{ADP}}\;+\;\frac{\left[Fru16BP\right]}{K_{Fru16BP}}\;+\;\frac{\left[ADP\right]\left[Fru16BP\right]}{K_{ADP}K_{Fru16BP}}\;+\;1}\right)) \end{array}$$

The ALDO rate equation was the reversible Uni-Bi random Michaelis-Menten equation (Equation 8) in all kinetic models as was reported in previous kinetic models (Marín-Hernández et al., 2011, 2014).

(8) $$v=\frac{Vmf\frac{\left[Fru1,6BP\right]}{K_{fru1,6bp}}-Vmr\frac{\left[DHAP\right]\left[G3P\right]}{K_{DHAP}K_{G3P}}}{1\;+\;\frac{\left[Fru1,6BP\right]}{K_{fru1,6bp}}\;+\;\frac{\left[DHAP\right]}{K_{DHAP}}+\;\frac{\left[G3P\right]}{K_{G3P}}\;+\;\frac{\left[DHAP\right]\left[G3P\right]}{K_{DHAP}K_{G3P}}}$$

Kinetics for TPI in the AS-30D model was here depicted by a mono-substrate simple reversible Michaelis-Menten equation (Equation 9) with mixed type inhibition by Fru1,6BP as it was previously determined (Moreno-Sánchez et al., 2016):

(9) $$v=\frac{Vmf\frac{\left[S\right]}{K_{s}\left(1\;+\;\frac{\left[I\right]}{Ki}\right)}\;-\;Vmr\frac{\left[P\right]}{Kp\left(1\;+\;\frac{\left[I\right]}{Ki}\right)}}{1\;+\;\frac{\left[S\right]}{K_{s}\left(\frac{1\;+\;\frac{\left[I\right]}{Ki}}{1\;+\;\frac{\left[I\right]}{\propto Ki}}\right)}\;+\;\frac{\left[P\right]}{K_{p}\left(\frac{1\;+\;\frac{\left[I\right]}{Ki}}{1\;+\;\frac{\left[I\right]}{\propto Ki}}\right)}}$$

α value is the factor by which *Ks* and *Kp* change when Fru1,6BP is bound to the enzyme; *Ki* is the inhibition constant for Fru1,6BP (*Ki*_Fru1, 6BP_).

In the HeLa model, the TPI rate equation (Equation 10) was a mono-substrate simple reversible Michaelis-Menten equation as it was previously determined (Marín-Hernández et al., 2011, 2014) with no inhibitors:

(10) $$v=\frac{Vmf\frac{\left[S\right]}{Ks}-Vmr\frac{\left[P\right]}{Kp}}{1+\frac{\left[S\right]}{Ks}+\frac{\left[P\right]}{Kp}}$$

GAPDH kinetics in the AS-30D model was here described by a simplified ordered Ter-Bi Michaelis-Menten equation (Equation 11) with mixed type inhibition by Fru1,6BP as previously determined (Moreno-Sánchez et al., 2016):

(11) $$v=\frac{Vmf\frac{\left[A\right]\left[B\right]\left[C\right]}{KaKbKc}\;-\;Vmr\frac{\left[P\right]\left[Q\right]}{KpKq}}{1\;+\;\frac{\left[A\right]}{Ka}\;+\;\frac{\left[A\right]\left[B\right]}{KaKb}\;+\;\frac{\left[A\right]\left[B\right]\left[C\right]}{KaKbKc}\;+\;\frac{\left[P\right]\left[Q\right]}{KpKq}\;+\;\frac{\left[Q\right]}{Kq}\;+\;\frac{\left[I\right]}{Ki}\;+\;\frac{\left[A\right]\left[B\right]\left[I\right]}{KaKb\propto Ki}\;+\;\frac{\left[A\right]\left[B\right]\left[C\right]\left[I\right]}{KaKbKc\propto Ki}\;+\;\frac{\left[P\right]\left[Q\right]\left[I\right]}{KpKq\propto Ki}}$$

where *A* = [NAD^+^], *B* = [G3P], *C* = [Pi], *P* = [BPG], *Q* = [NADH] with their respective affinity constants (*Ka, Kb, Kc, Kp*, and *Kq*). *Ki* is the inhibition constant for Fru1,6BP (*Ki*_Fru1, 6BP_). α value is the factor by which *Kb* (*Km*_G3P_) changes when Fru1,6BP is bound to the enzyme.

In the HeLa model, the GAPDH rate equation (Equation 12) was a simplified ordered Ter-Bi Michaelis-Menten equation as was previously determined and used in previous models (Marín-Hernández et al., 2011, 2014).

(12) $$v=\frac{Vmf\frac{\left[A\right]\left[B\right]\left[C\right]}{KaKbKc}\;-\;Vmr\frac{\left[P\right]\left[Q\right]}{KpKq}}{1\;+\;\frac{\left[A\right]}{Ka}\;+\;\frac{\left[A\right]\left[B\right]}{KaKb}\;+\;\frac{\left[A\right]\left[B\right]\left[C\right]}{KaKbKc}\;+\;\frac{\left[P\right]\left[Q\right]}{KpKq}\;+\;\frac{\left[Q\right]}{Kq}}$$

In all models the rate equations for PGAM and ENO were depicted by mono-substrate simple reversible Michaelis-Menten equation (Equation 13):

(13) $$v=\frac{Vmf\frac{\left[S\right]}{Ks}-Vmr\frac{\left[P\right]}{Kp}}{1+\frac{\left[S\right]}{Ks}+\frac{\left[P\right]}{Kp}}$$

in which [*S*] and [*P*] are the respective concentrations of substrates and products and their respective *Km* values (*Ks* and *Kp*) which were experimentally determined in previous works (Marín-Hernández et al., 2011, 2014).

The PYK rate equation (Equation 14) in all models was defined as simple random-bisubstrate Michaelis-Menten that represents the kinetics of the prevalent PYK isoform in cancer cells with no cooperativity or allosteric modulation by typical metabolites, as experimentally determined for AS-30D cells (Marín-Hernández et al., 2014; Moreno-Sánchez et al., 2016). *A, B, P*, and *Q* are PEP, ADP, Pyr, and ATP, respectively; *Ka, Kb, Kp*, and *Kq* are the *Km* values for the corresponding substrates and products.

(14) $$v=\frac{\frac{Vmf}{KaKb}\left(\left[A\right]\left[B\right]-\frac{\left[P\right]\left[Q\right]}{Keq}\right)}{1\text{​}+\text{​}\frac{\left[A\right]}{Ka}\text{​}+\text{​}\frac{\left[B\right]}{Kb}\text{​}+\text{​}\frac{\left[A\right]\left[B\right]}{KaKb}\text{​}+\text{​}\frac{\left[P\right]}{Kp}\text{​}+\text{​}\frac{\left[Q\right]}{Kq}\text{​}+\text{​}\frac{\left[P\right]\left[Q\right]}{KpKq}\text{​}+\text{​}\frac{\left[A\right]\left[Q\right]}{KaKq}\text{​}+\text{​}\frac{\left[P\right]\left[B\right]}{KpKb}}$$

In all models, the rate equations (Equation 15) for PGK and LDH were defined by random Bi-Bi reversible Michaelis-Menten for non-interacting substrates (α and β = 1) according to the reported literature (Marín-Hernández et al., 2011, 2014; Moreno-Sánchez et al., 2016); *Ka, Kb, Kp*, and *Kq* are the *Km* values for the corresponding substrates and products previously determined (Marín-Hernández et al., 2011, 2014; Moreno-Sánchez et al., 2016). In the case of PGK, *A, B, P*, and *Q* are 1,3BPG, ADP, 3PG, and ATP, respectively whereas for LDH they are NADH, Pyr, Lactate and NAD^+^, respectively.

(15) $$v=\frac{Vmf\frac{\left[A\right]\left[B\right]}{\alpha KaKb}-Vmr\frac{\left[P\right]\left[Q\right]}{\beta KpKq}}{1+\frac{\left[A\right]}{Ka}+\frac{\left[B\right]}{Kb}+\frac{\left[A\right]\left[B\right]}{\alpha KaKb}+\frac{\left[P\right]\left[Q\right]}{\beta KpKq}+\frac{\left[P\right]}{Kp}+\frac{\left[Q\right]}{Kq}}$$

In the HeLa model the rate-equation (Equation 16) for the monocarboxylate transporter (MCT) activity was incorporated (Marín-Hernández et al., 2014) since it catalyzes the expulsion of lactate and H+. With the inclusion of this reaction, it can be visualized why HeLa cell cultures become rapidly acidic. This was a mono-substrate reversible Michaelis-Menten equation in which Lac_in_ and Lac_out_ are the intra- and extra-cellular lactate concentrations; *K*_*Lacin*_ *and K*_*Lacout*_ are the *Km* values for the intra and extracellular lactate; *K*eq is the equilibrium constant of the reaction. The equation only included lactate as a ligand because kinetic parameters for the proton are not available.

(16) $$v=\frac{Vmf\left(\left[Lac_{in}\right]-\frac{\left[Lac_{out}\right]}{Keq}\right)}{K_{Lacin}\left(1+\frac{\left[Lac_{out}\right]}{K_{Lacout}}\right)+Lac_{in}}$$

### Molecular docking analysis

Crystal structures of human HK type I, TPI and GAPDH, and mouse HPI, were obtained from the protein data bank (accession numbers 4FOI, 1HTI and 3PFW, and 1U0F, respectively). The three dimensional models of the ligands used in the study were obtained from different crystal structures found in the protein data bank or downloaded from PubChem (https://pubchem.ncbi.nlm.nih.gov/). The models were optimized using the ArgusLab 4.0.1 (Planaria Software LLC, Seattle, WA) available at: http://www.arguslab.com and Maestro, version 9.1 (Schrodinger, LLC, New York, NY, 2010) softwares. For docking analysis, the ligands from the above protein crystal structures were removed using the software UCSF Chimera package 1.6 (Resource for Biocomputing, Visualization, and Informatics at the University of California, San Francisco, CA; supported by NIH P41 RR001081; Pettersen et al., 2004). The protein structure and ligand models were prepared for docking using the software ADT 1.5.2 (Sanner, 1999; Morris et al., 2009). Docking analysis of the enzyme structures and ligands were carried out using the software Autodock 4.2.5.1 (Huey et al., 2007) available at http://autodock.scripps.edu/. After docking, 100 conformations for each ligand were obtained, and then clustered for analysis using ADT 1.5.2 software. The conformations selected corresponded to the lowest values of binding energy and *Ki*. Analysis of the resulting structures and generation of the figures were achieved with PyMOL (The PyMOL Molecular Graphics System, Version 1.2.r1, Schrodinger, LLC).

## Results

### Effect of metabolic inhibition of low flux–controlling enzymes in the pathway flux

Inhibition of a low- or non-flux controlling step might be regarded as a mishap or misleading option to decrease the pathway flux because it requires almost complete inhibition (a “pharmacological knock out” or >80% inhibition) of the activity to decrease the pathway flux to the levels reached by inhibiting a flux-controlling step. However, there are experimental evidences indicating that oxamate and iodoacetate, presumed specific inhibitors of LDH and GAPDH, i.e., two non-controlling glycolytic enzymes, do affect the glycolytic flux of cancer, and non-cancer cells (Goldberg et al., 1965; Elwood, 1968; McKee et al., 1968; Coe and Strunk, 1970; Chatham et al., 1988; Moreno-Sánchez et al., 2016). Remarkably, this inhibition induces accumulation of Fru1,6BP and DHAP, being the former an inhibitor of HK (controlling enzyme), TPI, and GAPDH, whereas the latter is an inhibitor of HPI (another controlling enzyme; published data summarized in Supplementary Table 2).

The effect of oxamate on the activities of LDH, GAPDH, ENO, and PYK, which in turn affected the Fru1,6BP and DHAP concentrations (Moreno-Sánchez et al., 2016), was here *in silico* examined focusing on the concentration control coefficients for Fru1,6BP and DHAP by using the updated and modified kinetic models of AS-30D and HeLa glycolysis. These models were further refined (described in Section Kinetic Modeling) with respect to the models previously published (Marín-Hernández et al., 2014; Moreno-Sánchez et al., 2016). Enzymes that produce a metabolite have positive concentration control coefficients whereas those that consume it have negative ones (Fell, 1997). For glycolysis of AS-30D hepatocarcinoma cells, the model simulations indicated that GLUT, HK, and HPI have high positive concentration control coefficients values (from 1 to 2.3), whereas ENO (−0.57 and −0.99), PYK (−0.4 and −0.7), and GAPDH (−0.16 and −0.27) have high negative concentration control coefficients values on Fru1,6BP and DHAP, respectively (Table 1). For HeLa cells cultured for 24 h under different glucose concentrations (normo-, hypo-, or hyper-glucemic conditons) kinetic model simulations showed that GLUT, HK, and HPI also exert control on the synthesis of the same two metabolites, whereas GAPDH (−0.7 and −1.3) and PYK (−1.3 and −3) control their consumption (Table 1). Hence, in both types of cancer cells, the enzymes with high positive concentration control coefficients on Fru1,6BP and DHAP were those that also exerted the main control on the glycolytic flux (GLUT, HK, and HPI) and hence, their inhibition should decrease Fru1,6BP and DHAP concentrations. In contrast inhibition of GAPDH, ENO, and PYK, which have high negative concentration control coefficients should increase the levels of Fru1,6BP and DHAP. It is worth noting that without kinetic modeling it would not have been possible to unveiled the important role of GAPDH, ENO, and PYK on the indirect modulation of the flux-controlling enzymes by Fru1,6BP and DHAP. By the present *in silico* analysis, these other metabolic regulatory mechanisms of cancer glycolysis became apparent and were further analyzed.

**Table 1:** **Concentration control coefficients of glycolytic steps on Fru1,6BP, and DHAP obtained *in silico* using the updated kinetic models of glycolysis in AS-30D and HeLa cells (see Section Materials and Methods) under normoxia (20% O_2_)**.

**Enzymes**	**AS-30D cells**		**HeLa cells**					
			**Hyperglycemia**		**Normoglycemia**		**Hypoglycemia**	
	**Fru1,6BP**	**DHAP**	**Fru1,6BP**	**DHAP**	**Fru1,6BP**	**DHAP**	**Fru1,6BP**	**DHAP**
GLUT	1.9	1	0.66	0.36	0.72	0.4	2.5	1.5
HK	2.3	1.3	2.3	1.3	2.4	1.4	1.9	1.1
HPI	1.9	1.1	1.5	0.8	1.3	0.7	0.8	0.4
PFK1	0.2	0.1	0.7	0.4	0.9	0.5	0.7	0.4
ALDO	−0.4	−0.003	−0.3	0.0007	−0.4	−0.0007	−0.6	0.001
TPI	−0.06	−0.06	−0.004	−0.004	−0.004	−0.004	−0.008	−0.008
GAPDH	−0.27	−0.16	−1.3	−0.7	−1.2	−0.7	−1.2	−0.7
PGK	−0.1	−0.08	−0.01	−0.008	−0.02	0.01	−0.02	−0.01
PGAM	−0.09	−0.05	−0.05	−0.03	−0.06	−0.03	−0.07	−0.04
ENO	−0.99	−0.57	−0.06	−0.03	−0.06	−0.03	−0.07	−0.05
PYK	−0.7	−0.4	−3	−1.7	−2.5	−1.5	−2.0	−1.3
LDH	−0.04	−0.02	−0.03	−0.02	−0.02	−0.01	−0.03	−0.02
MCT			−0.2	−0.1	−0.2	−0.1	−0.1	−0.07

**Figure 1:**
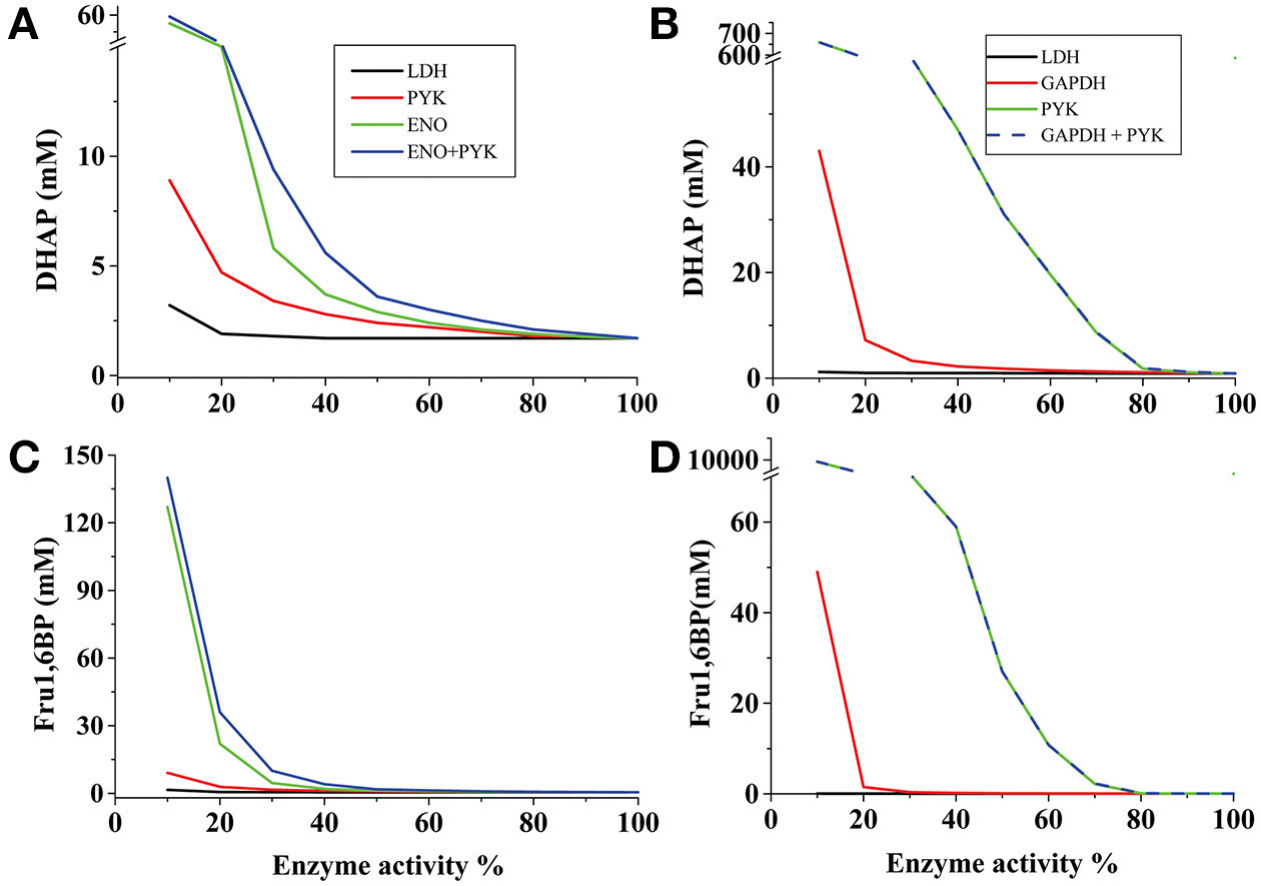
**Dependence of Fru1,6BP and DHAP concentrations on the activity of different pathway enzymes of cancer glycolysis**. The reference 100% enzyme activity values were those corresponding to the respective *Vmax* values for the forward reaction whereas Fru1,6BP and DHAP concentrations were those predicted by each model (**A,C** for AS-30D cells; **B,D** for hypoglycemic HeLa cells). When two enzymes were simultaneously titrated, identical variation in the activities was applied. In the case of LDH, ENO, and GAPDH, a decrease of the *Vmaxf* value was accompanied by a proportional decrease in the *Vmaxr* value.

In the AS-30D kinetic model it was evaluated the effect of LDH, ENO or PYK inhibition on the levels of Fru1,6BP and DHAP. LDH showed low control on their concentrations (−0.4 and −0.02; Table 1) since an 80% decrease in its activity only induced a marginal increase in their concentrations (Figure 1); identical results were attained with PGK and PGAM (data not shown). In contrast, a similar inhibition of ENO and PYK activities led to marked accumulation of Fru1,6BP and DHAP (Figures 1A,C). Similar results were obtained with the HeLa model under hypoglycemia when modulation of the GAPDH, PYK, PGK, PGAM, and LDH activities were simulated (Figures 1B,D). These analyses strongly suggested that the increase in Fru1,6BP and DHAP levels reported in AS-30D, Ehrlich ascites, sarcoma 37 ascites and HeLa cells treated with oxamate (Goldberg et al., 1965; Elwood, 1968; Coe and Strunk, 1970; Moreno-Sánchez et al., 2016), was a consequence of ENO and PYK inhibition rather than of LDH inhibition, contrary to the most common interpretation.

**Figure 2:**
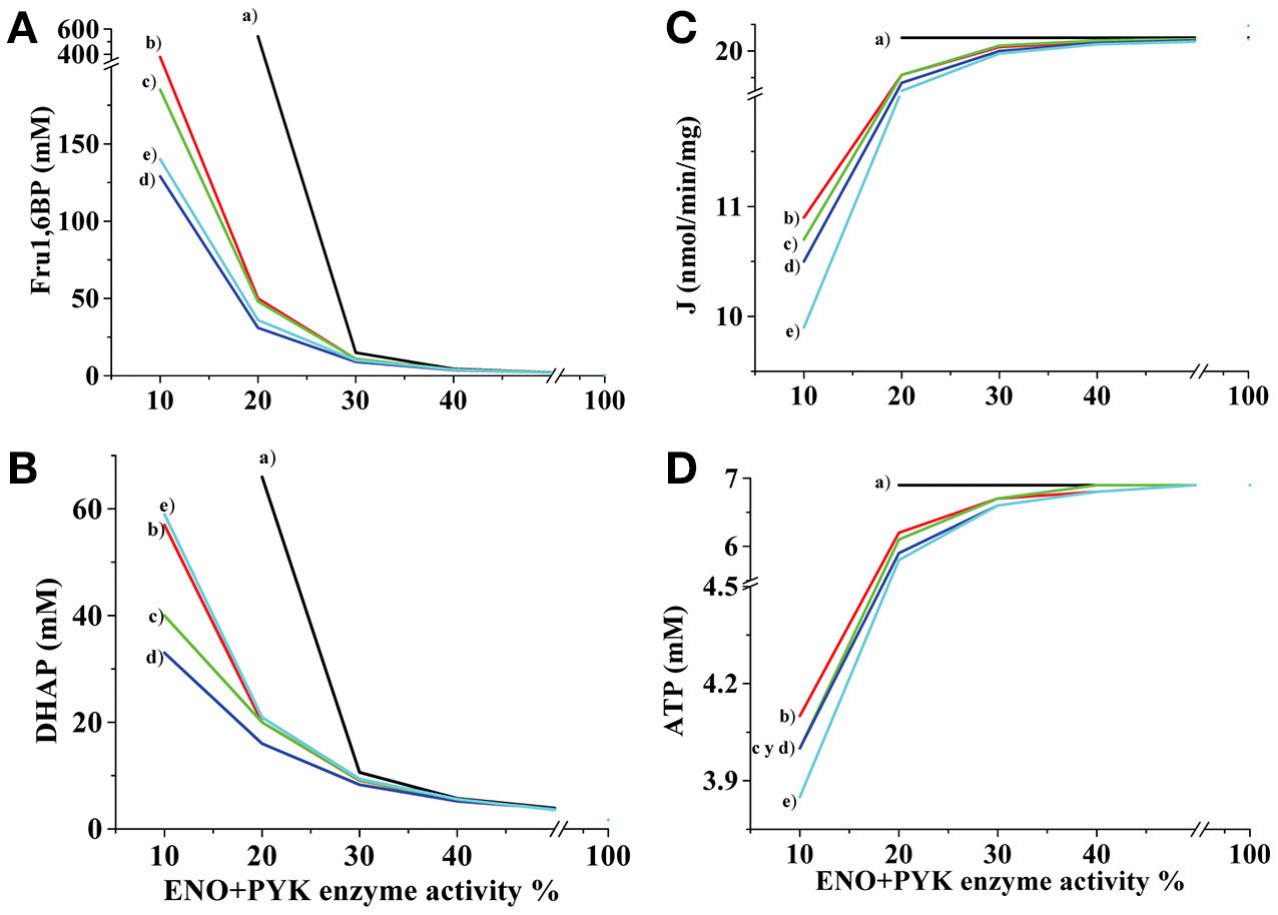
**Effect of ENO and PYK inhibition on Fru1,6BP, DHAP, and ATP concentrations and glycolytic flux**. The kinetic model for AS-30D glycolysis was used. ENO *plus* PYK activities were modulated and the effects on Fru1,6BP **(A)**, DHAP **(B)**, glycolytic flux **(C)**, and ATP **(D)** were determined. Several simulations were made with or without enzyme inhibition by Fru1,6BP and DHAP on no enzyme (a); on HPI (b); on HK (c); on both HPI and HK (d); and on HPI, HK, TPI, and GAPDH (e). The respective HPI, HK, TPI, and GAPDH rate equations with Fru1,6BP and DHAP inhibition are depicted in the Methods Section.

To further establish the influence in the glycolytic flux and ATP concentration of the inhibitory effect of Fru1,6BP and DHAP on flux-controlling (HK and HPI) and not flux-controlling (TPI and GADPH) enzymes, several simulations were made with the AS-30D model (Figure 2). When inhibition of Fru1,6BP and DHAP was not included in the rate equations of HK, HPI, TPI and GAPDH, the decrease of ENO and PYK activities still increased the levels of Fru1,6BP and DHAP but the concentration of ATP and glycolytic flux were not modified (Figure 2). A similar effect was observed when inhibition of Fru1,6BP on the TPI and GAPDH rate equations was included (data not shown). These last observations indicated that inhibition of ENO and PYK activities *per-se* was not sufficient to decrease the glycolytic flux. Only when the Fru1,6BP and DHAP inhibitions on the HPI and HK rate equations were included, the glycolytic flux and ATP concentration decreased (Figure 2).

Furthermore, the Fru1,6BP and DHAP levels indeed increased in cells treated with oxamate (reported by Moreno-Sánchez et al., 2016) or iodoacetate (Table 2). These experimentally determined metabolite concentrations were also closely simulated by kinetic modeling when decreasing by ~75% the ENO+PYK activities, and including the Fru1,6BP and DHAP inhibition of HK, or HPI, or both HK+HPI, or HPI+HK+TPI+GAPDH (Figure 2). Hence, the interactions of these metabolites with HK and HPI are apparently also involved in the mechanisms of control of their own intracellular levels. Incubation with oxamate or iodoacetate promoted a severe decrease (3.5–4.6 times vs. control) in the intracellular ATP (Table 2). Although cell viability remains high (>90%), it may be possible that these inhibitors also affect the mitochondrial function thus perturbing the cell ATP levels (Martin-Requero et al., 1986; Cano-Ramírez et al., 2012). Since a significant HK fraction in cancer cells is bound to mitochondria, OxPhos also provides ATP for this glycolytic reaction. However, this interplay between glycolysis and OxPhos through the ATP/ADP ratio has not been included in the present kinetic models because the subcellular distribution of the HK isoforms has not been determined under the different O_2_ and glucose culture conditions.

**Table 2:** **Fru1,6BP and DHAP concentrations in AS-30D cells treated with oxamate or iodoacetate**.

**Metabolite/flux**	**Control**	**Iodoacetate (mM)**		**Oxamate (mM)**	
		**2**	**4**	**10**	**20**
Glc6P	10 ± 5 (3)	3.9 ± 2 (4)	3.5 ± 2 (3)	4^a^	4^a^
Fru6P	2 ± 1 (3)	1.5 ± 0.3 (3)	1.6 ± 0.5 (3)	1.2^a^	1.3^a^
Fru1,6BP	2.4 ± 1.7 (3)	7 ± 1 (3)^*^	8 (2)^c^	23^a^	22^a^
DHAP	1.8 ± 0.4 (5)	33 ± 9 (3)^**^	17 (2)^c^	11^a^	11^a^
ATP	6 ± 2 (3)	1.4 ± 0.5 (3)^*^	1.3 ± 0.3 (3)^*^	1.7^a^	1.4^a^
Glycolytic flux	8 ± 3 (3)	2 ± 2 (4)^*^	1 (2)^c^	4.3^a^	4.2^a^
Methylglyoxal^b^	< 0.3 (4)^d^	2.2 ± 0.6 (3)	N.M.	N.M.	3.6 ± 1.6 (4)

Cells were incubated in Krebs-Ringer medium with no glucose added at 37°C for 1 h with the indicated inhibitor concentrations. Then, 5 mM glucose was added and the metabolite concentrations and glycolytic flux were determined after 10 min. Metabolites in mM. Fluxes in nmol/min^*^mg protein.

a For comparison, these values were taken from Moreno-Sánchez et al. (2016).

b Methylglyoxal in nmol/mg protein.

c The independent experiment values showed a 15% difference between them.

d The limit of methylglyoxal detection was ~0.3 nmoles.

Asterisks indicate statistically significant differences compared with control (

* P ≤ 0.05,

** P ≤ 0.0005) using Student's t-test for non-paired samples. The data shown represent the mean ± standard deviation with the number of independent preparations assayed between parentheses. N.M. not measured.

### Accumulation of toxic metabolites contributes to decrease cancer glycolysis

Another possible consequence of DHAP accumulation is the production of methylglyoxal, which can also inhibit the glycolytic flux in cancer and normal cells (Leoncini et al., 1989; Halder et al., 1993; Biswas et al., 1997). In this regard, cells treated with oxamate showed increased methylglyoxal levels (Table 2). Similarly, significant increases in Fru1,6BP, DHAP and methylglyoxal were observed in cells treated with iodoacetate (Table 2). This last inhibitor primarily affects GAPDH (Sabri and Ochs, 1971), which also exerts control on the concentrations of Fru1,6BP and DHAP (Table 1). In addition, in the iodoacetate-treated cells, significant decreases in the ATP concentrations and glycolytic flux were observed with respect to control cells, whereas the Glc6P and Fru6P levels did not change (Table 2). All these changes in metabolites and glycolytic flux were similar to those previously observed in cells treated with oxamate (Table 2; and Moreno-Sánchez et al., 2016). Therefore, it is suggested that iodoacetate induces glycolysis inhibition mainly through accumulation of Fru1,6BP and DHAP.

Methylglyoxal affects PYK and GAPDH activities (Leoncini et al., 1989; Halder et al., 1993; Biswas et al., 1997). However, in cells treated with iodoacetate (2 mM) or oxamate (20 mM) no changes were attained in these enzyme activities (data not shown). This discrepancy may be attributed to the high concentrations of methylglyoxal (2.5 mM) used in previous papers (Leoncini et al., 1989; Halder et al., 1993). Alternatively, the glyoxalase system in AS-30D cells might be highly efficient for methylglyoxal detoxification, a hypothesis that remains to be experimentally determined.

### Effect of the inhibition mechanism of HPI on pathway properties

In the previous sections it was shown that it is indeed possible to significantly inhibit glycolysis by affecting non-flux controlling enzymes. Traditionally, competitive inhibitors have been studied or designed for drug therapy. However, such type of inhibitors induces substrate accumulation which in turn eventually displaces the inhibitor from the enzyme binding site, attenuating its inhibitory impact. Therefore, it was interesting to test with the present kinetic model the effect of different types of inhibition mechanisms on the pathway flux. This is relevant because the experimental results above showed accumulation of metabolites that affect the activities of the main controlling enzymes. Therefore, the kinetics of a flux-controlling step such as HPI was analyzed. This enzyme catalyzes a monosubstrate reaction and is strongly regulated by Fru1,6BP, Ery4P, and 6PG (Supplementary Table 2) which are competitive inhibitors (Marín-Hernández et al., 2011). In these simulations, DHAP was not included as HPI inhibitor because it only affects at high concentrations (*Ki*_DHAP_ = 9.4 mM and in HeLa cells physiological concentrations of DHAP are 0.5–0.8 mM; Marín-Hernández et al., 2014).

The first versions of the kinetic model of cancer glycolysis previously published predicted low levels of Glc6P and high glycolytic flux which were in disagreement with the experimental values. The model refinement process indicated that HPI activity should be inhibited to properly simulate the experimental values (Marín-Hernández et al., 2011). Thus, it was experimentally determined that physiological levels of Ery4P, 6PG, and Fru1,6BP competitively inhibited HPI activity vs. Fru6P or Glc6P (Marín-Hernández et al., 2011; Moreno-Sánchez et al., 2016; published data summarize in Supplementary Table 2). Then, multiple competitive-type inhibition was incorporated in the HPI rate-equation to accurately simulate the experimental Glc6P concentrations and glycolytic flux (Marín-Hernández et al., 2011). Here it was now explored the effect that different types of HPI inhibition have on pathway metabolite concentrations and flux to establish which kind of inhibitor is more efficient in blocking controlling steps and glycolytic flux.

**Figure 3:**
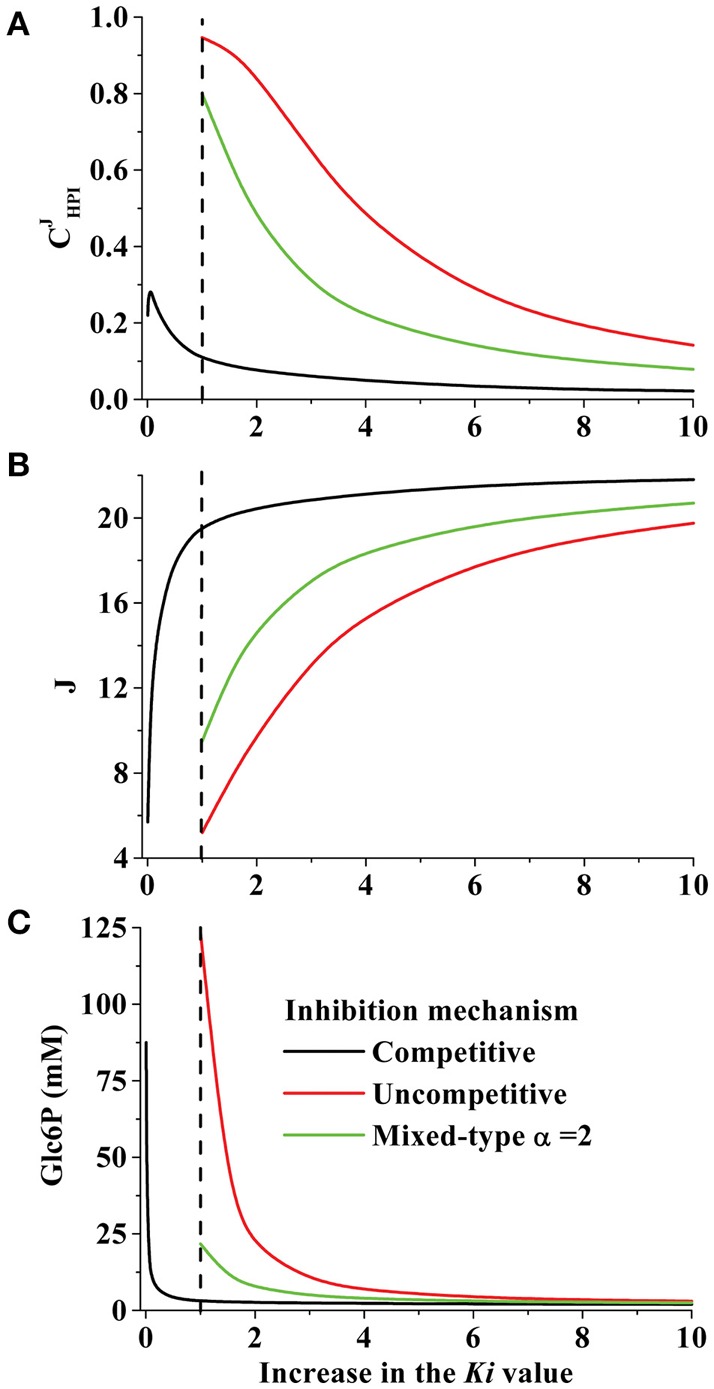
**Effect of changing the *Ki* value and inhibition mechanism of tumor HPI on (A) HPI flux control coefficient; $C_{HPI}^{J}$, (B) pathway flux and (C) Glc6P concentration**. Using the kinetic model for hypoglycemic HeLa cells, increasing *Ki* values for Ery4P, Fru1,6BP, and 6PG were simulated with competitive, uncompetitive or mixed type inhibition mechanisms. In the case of mixed type inhibition, α = 2. The dashed line indicates the normalized *Ki* value which was experimentally determined (*Ki*_Ery4P_ = 1 μM; *Ki*_Fru1, 6BP_ = 60 μM; *Ki*_6PG_ = 15 μM).

For competitive inhibition, the effect of changing the *affinities* (1/*Ki*) of HPI inhibitors was modeled. A decrease of 90% in the *Ki* value (i.e., the affinity values for inhibitors were increased by 10-fold) increased the HPI flux-control coefficient to a value of 0.27 (Figure 3A). The reason for this behavior was that accumulation of Glc6P (Figure 3C) attenuated the binding of the physiological inhibitors to HPI and also exerted strong inhibition on HK. Furthermore, the HPI flux control remained unchanged (0.25) when the *Ki* value was decreased 100-fold (*Ki* = 0.01; Figure 3A), but the glycolytic flux drastically decreased as a consequence of HK inhibition by accumulated Glc6P.

When HPI inhibitors were all considered as uncompetitive or mixed type inhibitors in the kinetic model of hypoglycemic HeLa cells, it was necessary to decrease their affinities by 6–10 times (i.e., their *Ki* values were increased 6–10) to keep unaltered the HPI flux-control coefficient, pathway flux and Glc6P concentration (Figures 3A–C, respectively). However, it was noted that with uncompetitive inhibition, an increase in the *K*i values by only three-fold yielded a high flux control coefficient of 0.65 with concomitant remarkable suppression of pathway flux and accumulation of Glc6P. This enhanced accumulation of substrates of the inhibited enzyme by uncompetitive inhibitors (vs. competitive inhibitors) was envisioned three decades ago (Cornish-Bowden, 1986), but perhaps because examples of uncompetitive inhibition have not been profusely found, studies on this issue have not been developed. With mixed-type inhibition, the three-fold increase in *Ki* values brought about milder effects on HPI flux control, pathway flux and Glc6P concentration.

These *in silico* simulations suggested that both uncompetitive and mixed-type inhibition can perturb the pathway flux, at a significantly greater extent than competitive inhibition, because these types of inhibition affect *Vmax* (which is not altered by competitive inhibitors) and catalytic efficiency (*Vmax/Km*). It is recall that the *Vmax* value is directly linked to the content of active enzyme (*Vmax* = *k*_*cat*_ × [enzyme]_total_) and hence to transcriptional/translational regulation and protein degradation. The design of new inhibitors should consider the uncompetitive and mixed-type inhibition mechanisms to generate potent drugs against cancer glycolysis.

### Docking analysis predicts potency of regulatory metabolites of HPI

**Figure 4:**
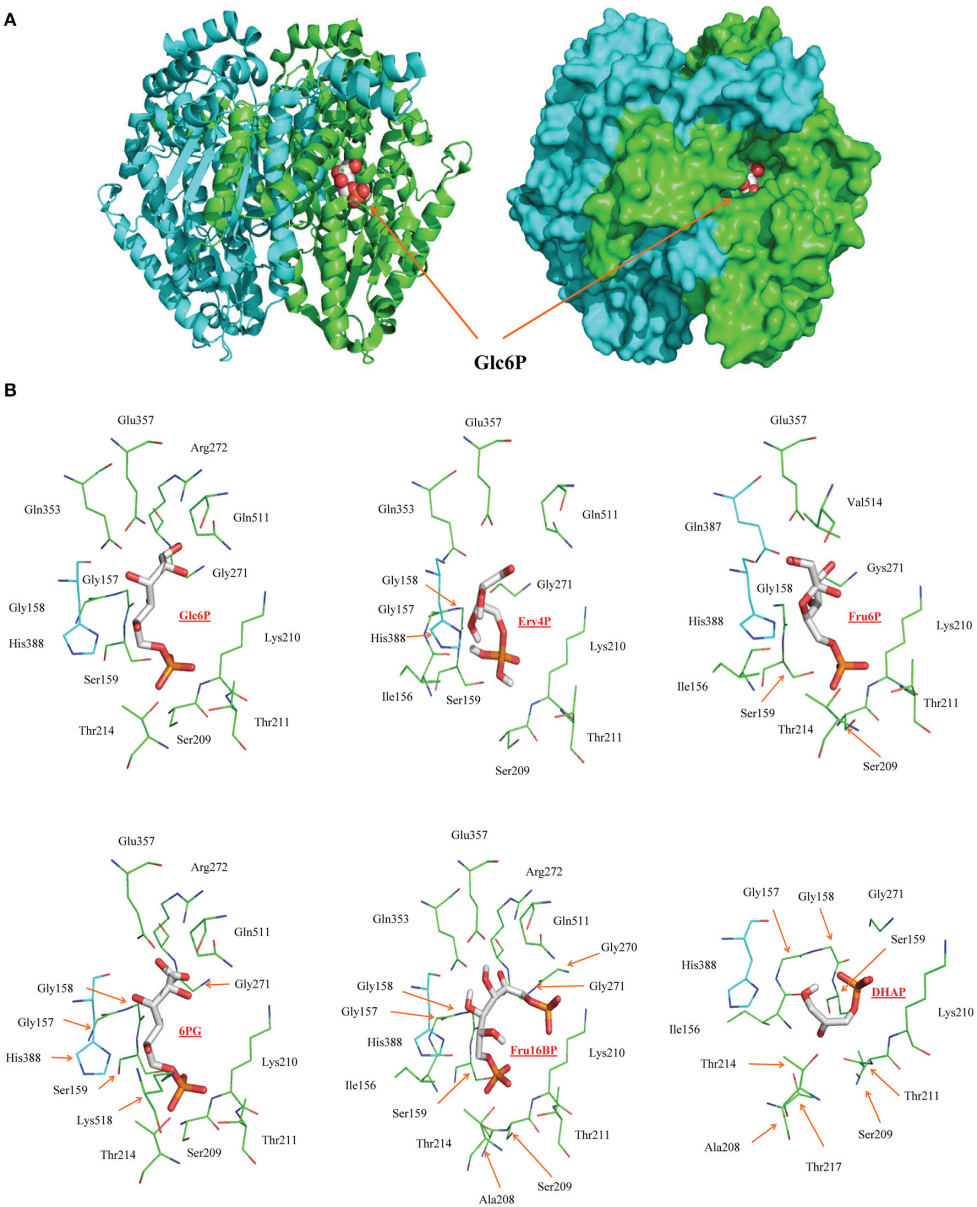
**Docking of metabolic inhibitors into mouse hexose phosphate isomerase active site**. **(A)** Structure of the dimer of mouse HPI showing the substrate Glc6P (red spheres) bound to one of the active sites. The active site of each monomer is formed by a cleft between two domains at the dimer interface. **(B)** Backbone representation of the residues located within 4 Å of the ligands (shown as sticks) bound in the active site. Residues colored cyan belong to subunit B of the dimer.

To explore why regulatory metabolites may have different potencies, a docking analysis of metabolic inhibitors was performed on HPI. This analysis showed that the HPI competitive inhibitors can indeed adequately bind to the substrate binding site and be stabilized by the same amino acids in the active site (Figure 4). The binding energies were −5.62 (Ery4P), −4.57 (6PG), −3.63 (Fru1,6BP), and −2.64 (DHAP) Kcal/mol. The estimated *Ki* values (in mM) were 0.076 (Ery4P), 0.45 (6PG), 2.2 (Fru1,6BP), and 11.6 (DHAP) which indicated that Ery4P and 6PG bind more tightly to the enzyme active site compared with the other two metabolites. These results correlated with previous data indicating that the most potent HPI inhibitors are Ery4P (*Ki* = 0.8–2.5 μM) and 6PG (*Ki* = 6.8–18 μM; Marín-Hernández et al., 2011). The discrepancy between the theoretical and experimental *Ki* values may be due to limitations in the docking procedure since for the analysis, the enzyme structures were considered rigid whereas only the ligands were flexible (for the limitation in the number of rotating bonds that can be assigned). However, enzyme structures are flexible and upon substrate or modifier binding, the active sites have in general conformational changes that in most cases favor tighter ligand coupling. However, despite these limitations, the docking data analysis predicted the order of binding efficiency and potency of the HPI inhibitors. Also, docking analysis showed that Fru1,6BP can bind to the active sites of HK, TPI, and GAPDH (Supplementary Figure 1).

## Discussion

### Feedback inhibition by glycolytic intermediaries on flux controlling enzymes

A recent study by our group (Moreno-Sánchez et al., 2016) showed that oxamate inhibition of cancer glycolysis was mediated by the direct moderate inhibition of several pathway sites such as LDH, PYK, and ENO. The simultaneous oxamate inhibition of these non-controlling enzymes induced the accumulation of Fru1,6BP and DHAP, which behaved as inhibitors of HK, HPI, TPI, and GAPDH. In the present study, it was shown that inhibition of down-stream non-controlling enzymes may affect pathway flux only if Fru1,6BP and DHAP are accumulated. It was also shown that the mechanistic basis of this glycolysis suppression was to specifically block the steps with predominant control on the Fru1,6BP and DHAP concentrations, which were PYK, ENO, and GAPDH, but not LDH or PGK. Perturbation of other pathways by inhibiting non-controlling steps may occur as long as the ensuing accumulation of metabolites affects the activities of the main controlling steps. For instance, inhibition of malate dehydrogenase, a Krebs cycle non-controlling step, brings about accumulation of NAD^+^, malate, fumarate, and succinate. And high levels of these metabolites may alter the activities of isocitrate and 2-oxoglutarate dehydrogenases, the main controlling steps of Krebs cycle.

Although iodoacetate is an unspecific drug that may covalently alkylates thiol groups at the active sites of many enzymes and hence may show toxicity, treatment of Ehrlich ascites carcinoma-bearing mice with iodoacetate significantly increases the median cumulative survival time and percentage of survivors, as well as decreases the tumor size (Fahim et al., 2003). Similarly, oxamate is able to inhibit the chondrosarcoma and nasopharyngeal carcinoma growth in nude mice (Li et al., 2013; Hua et al., 2014). It should be noted that the observed improvement in tumor-bearing animals treated with these compounds is the result of several combined processes including abolishment of tumor glycolysis and activation of several rescue pathways such as immune system (Rheins et al., 1975) and antioxidant defense (Fahim et al., 2003).

Although the drugs tested (oxamate and iodoacetate) might have similar effects on non-cancer cells, both inhibitors are well-tolerated in animals and human non-cancer cell lines, suggesting that normal cells are less sensitive to glycolysis inhibition, likely due to a lower dependence on glycolysis for their proliferation with respect to tumor cells. In addition, glycolysis inhibition of tumor associated fibroblasts (reverse Warburg effect) may be also beneficial to deter tumor growth (Pavlides et al., 2009; Martinez-Outschoorn et al., 2011).

**Figure 5:**
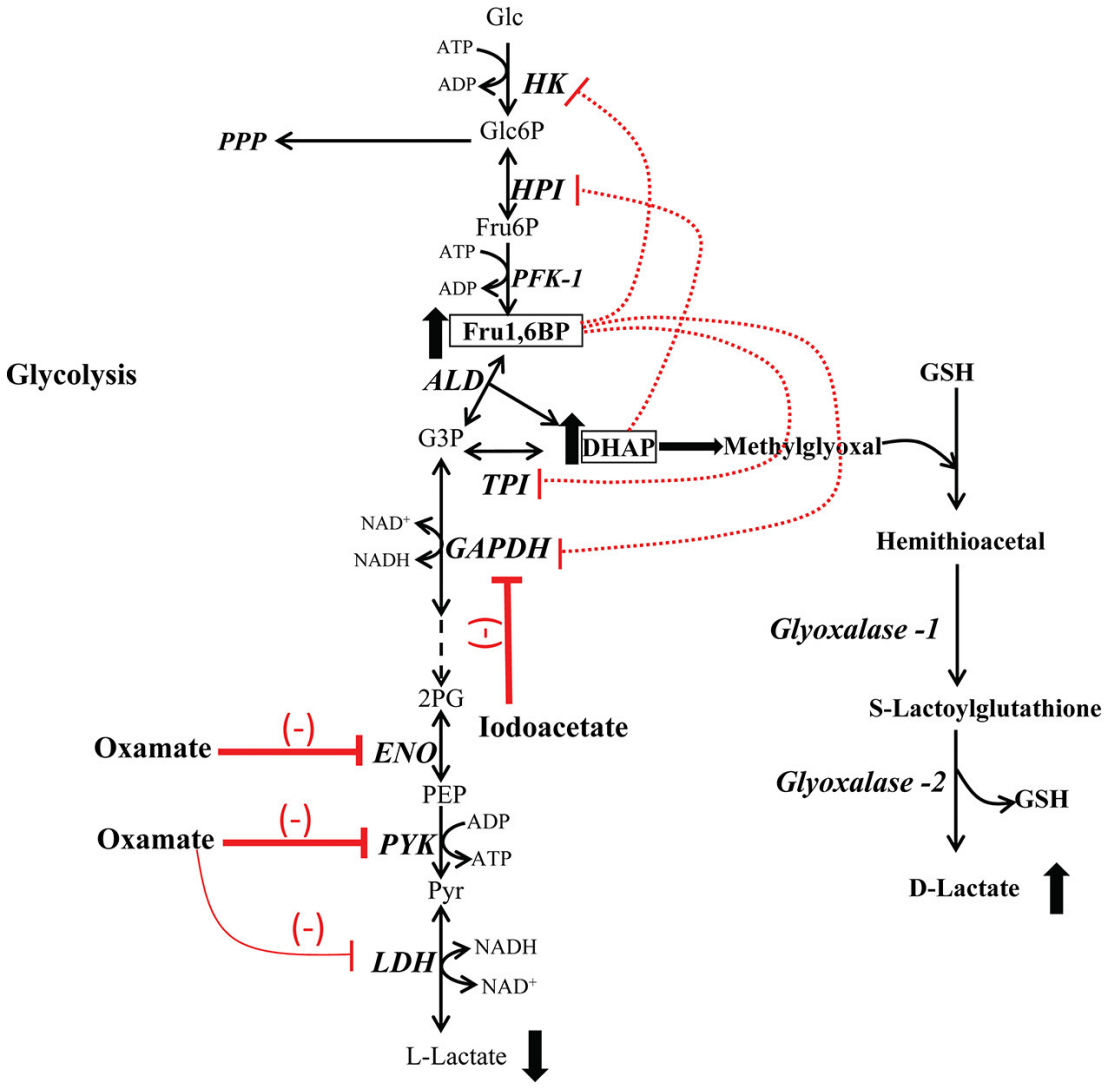
**Glycolysis inhibition by accumulation of fructose-1,6-bisphosphate and dihydroxyacetone phosphate**. Inhibition of ENO and PYK by oxamate, GAPDH inhibition by iodoacetate, induces accumulation of **Fru1,6BP** and **DHAP**, which behave as inhibitors of HK, HPI, GAPDH, and TPI at high concentrations, leading to a decrease in the L-lactate production (glycolysis inhibition). High levels of DHAP produce methylglyoxal that is converted in D-lactate by the glyoxalase system. PPP, pentose phosphate pathway.

As suggested by the data of the present study, the anticancer effect observed of these unspecific drugs (Fahim et al., 2003; Li et al., 2013; Hua et al., 2014) may be associated with the inhibition of glycolysis mediated by (i) the accumulation of Fru1,6BP and DHAP which in turn inhibit the main flux-controlling enzymes HK and HPI (Figure 5); and (ii) the accumulation of methyglyoxal. For cancer treatment, this mechanism may help in the design of new strategies to inhibit essential metabolic pathways such as OxPhos, the antioxidant system and anabolic routes.

Based on the *in silico* kinetic modeling analysis indicating that both uncompetitive and mixed-type inhibitors can perturb at a significantly greater extent the pathway flux and metabolite concentrations than competitive inhibitors, it is concluded that elevation of the Fru1,6BP levels will have a more severe depressing effect on the glycolytic flux of cancer cells than that of DHAP levels because the former behaves as a mixed-type inhibitor of HK whereas the latter competitively inhibits HPI (Moreno-Sánchez et al., 2016).

### Synthesis of toxic metabolites for cancer glycolysis

Fru1,6BP is a product of the PFK-1 reaction and a weak inhibitor of HK, HPI, GAPDH, and TPI (Marín-Hernández et al., 2011; Moreno-Sánchez et al., 2016). In addition of being an activator of PYKM2, Fru1,6BP may indirectly inhibit oxidative phosphorylation (Mazurek et al., 2002; Díaz-Ruiz et al., 2008). On the other hand, DHAP is one of the two products of Fru1,6BP breakdown, it is a weak HPI inhibitor (Moreno-Sánchez et al., 2016) and together with glyceraldehyde-3- phosphate represent the most important endogenous source of methylglyoxal (Allaman et al., 2015). The latter compound is one of the most potent glycating agents naturally produced within cells; it reacts with proteins, lipids and nucleic acids to form advanced glycation end products (AGEs; Allaman et al., 2015). High levels of this metabolite can be reached when the concentrations of their precursors are elevated, such as in impaired glucose utilization and TPI deficiency (Ahmed et al., 2003). The glyoxylase system is the main ubiquitous pathway for methylglyoxal detoxification (Figure 5) and is involved in tumor development, growth, migration, apoptotic evasion, and multidrug resistance. Increased levels and activities of glyoxylases 1 and 2 in diverse types of cancer (bladder, breast, colon, lung, and prostate) have been observed (Thornalley and Rabbani, 2011; Geng et al., 2014). Therefore, they have been considered as malignancy biomarker and potential anti-cancer target.

One attractive novel approach for targeting cancer cells, derived from the present study, which deserves further experimental assessment, is the use of inhibitors of GAPDH, ENO, and PYK together with glyoxylase inhibitors, which at relatively low doses do not perturb host homeostasis. This particular multi-drug treatment would induce DHAP accumulation which in turn would lead to enhanced levels of methylglyoxal, severely compromising cancer cell growth and viability. Although, methylglyoxal has several toxic effects, it has shown anticancer activity in tumor-bearing mice and slight side-effects (Ghosh et al., 2006). Furthermore, high concentrations of methylglyoxal (2–7.5 mM) strongly inhibits OxPhos and glycolysis, drastically decreasing the ATP level in cancer cells and apparently showing no effect on normal cells and tissues (Ray et al., 1991; Biswas et al., 1997). In tumor-bearing animals similar high levels of methylglyoxal concentrations in blood (13–19 mM) had no apparent toxic effect on vital organs (liver, kidney) but increased their life span by inhibiting tumor growth (Ghosh et al., 2006). This therapeutically exciting difference has been attributed to alterations in complex I and GAPDH of tumor cells that increase sensitive to methylglyoxal with respect to non-tumor enzymes (Biswas et al., 1997; Ray et al., 1997). Other reports indicate that methylglyoxal (30 μM) inhibits complex III and ATP synthesis in vascular smooth A-10 cells (Wang et al., 2009).

### Uncompetitive inhibition is the most potent mechanism to block glycolytic flux

There are three mechanisms by which a reversible inhibitor may interact with an enzyme: competitive, uncompetitive, and mixed type inhibition (Segel, 1975); the non-competitive inhibition should be considered as a special, non-common case of mixed-type inhibition. A molecule that is structurally similar to the natural substrate may reversible bind to the enzyme active site and act as a competitive inhibitor. In this regard, docking simulations were performed to support this assumption for HPI since the regulatory metabolites Fru1,6BP, Ery4P, 6PG, and DHAP readily bind to the substrate binding site and are stabilized by the same amino acids involved in the Glc6P and Fru6P binding (Figure 4). Thus, competitive inhibitors are common in metabolic pathways because the products of each reaction and several other pathway intermediaries have structural similarity with the substrate. As a consequence, Fru1,6BP, Ery4P, and 6PG behave as competitive inhibitors of HPI vs. the substrate Glc6P and product Fru6P, regulating the supply of Glc6P for pentose phosphate and glycogen synthesis pathways.

Nevertheless, competitive inhibitors can be also readily displaced from the active site by high substrate concentrations, thereby restoring enzyme activity. Thus, the physiological effect of competitive inhibitors is to provide an immediate response of the targeted enzyme/transporter which will be attenuated in the medium term. Then, although competitive inhibitors are easier to find in nature or be designed and manufactured, they are not pharmacologically efficient drugs.

In contrast, the effects of the uncompetitive and mixed-type inhibitors cannot be overcome by increasing the substrate concentration; in fact, for uncompetitive inhibition it becomes more significant at increasing substrate concentrations (Cornish-Bowden, 1986). Using a kinetic model of the parasite *Trypanosoma brucei* glycolysis it was concluded that inhibition of the pyruvate transport would be more effective for perturbing the pathway with an uncompetitive inhibitor (followed by mixed-type) than with a competitive one (Eisenthal and Cornish-Bowden, 1998). Although, uncompetitive inhibitors are not common, there are recent reports about the identification of uncompetitive inhibitors of human γ-glutamyl transpeptidase and P-glycoprotein, proteins that can play an important role in drug-resistance in cancer (Wickham et al., 2013; Teng et al., 2015).

Uncompetitive and mixed-type inhibitions modify the *Vmax* value, which is a kinetic parameter that has a strong influence on the degree of control that each pathway enzyme exerts (Marín-Hernández et al., 2014). GLUT was the main controlling step of glycolysis in HeLa hypoglycemic cells and *T. brucei* because its activity (i.e., *Vmax*) was the lowest (Bakker et al., 1999; Marín-Hernández et al., 2014). In contrast, PFK-I has low activity in tumor cells but it has no control on the pathway flux because Fru2,6BP activation increases several-fold its activity (Moreno-Sánchez et al., 2012).

## Conclusion

Kinetic modeling studies have shown that only the simultaneous inhibition of several flux-controlling steps will have significant impact on glycolytic flux and ATP concentration in cancer cells. This can be accomplished by direct inhibition using, preferentially, uncompetitive specific drugs or indirectly through the accumulation of regulatory metabolites of the flux-controlling steps by inhibiting enzymes that exert low flux-control.

## Author contributions

AM-H, IDM-M, and JSR-Z performed the *in vitro* and *in silico* experiments and docking analysis, AM-H, SR-E, RM-S, and ES planned experiments, analyzed data, contributed reagents, and wrote the paper.

### Conflict of interest statement

The authors declare that the research was conducted in the absence of any commercial or financial relationships that could be construed as a potential conflict of interest.

## Abbreviations

DHAP: dihydroxyacetone-phosphate
ENO: enolase
Ery4P: erythrose-4-phosphate
Fru1, 6BP: fructose-1,6-biphosphate
GAPDH: glyceraldehyde 3-phosphate dehydrogenase
Glc6P: glucose 6-phosphate
GLUT: glucose transporter
HK: hexokinase
HPI: hexosephosphate isomerase
LDH: lactate dehydrogenase
6PG: 6-phosphogluconate
PYK: pyruvate kinase
TPI: triosephosphate isomerase.
