# Supplementary material for: Inhibition of Non-flux-Controlling Enzymes Deters Cancer Glycolysis by Accumulation of Regulatory Metabolites of Controlling Steps
Source: Front Physiol. 2016 Sep 23;7:412. doi: 10.3389/fphys.2016.00412 (PMC5033973; doi:10.3389/fphys.2016.00412)
Supplement: Supplementary file 1 [file Table1.DOCX]

Supplementary Material

Inhibition of non flux-controlling enzymes deters cancer glycolysis by accumulation of regulatory metabolites of controlling steps

**Álvaro Marín-Hernández*, José Salud Rodríguez-Zavala, Isis Del Mazo-Monsalvo, Sara Rodríguez-Enríquez, Rafael Moreno-Sánchez and Emma Saavedra***

*** Correspondence:** Álvaro Marín Hernández, Ph. D. and Emma Saavedra, Ph. D. e-mail: emma_saavedra2002@yahoo.com; marinhernndez@yahoo.com.mxmarinhernndezq@yahoo.com.mx; alvaro.marin@cardiologia.org.mx

**Supplementary Table 1. Summary of the kinetic parameter values used in GEPASI for kinetic models of glycolysis in AS-30D and HeLa cancer cells**

| Enzyme |  | AS-30D | HeLa | | | |
| --- | --- | --- | --- | --- | --- | --- |
|  |  |  |  | Hyperglycemia | Normoglycemia | Hypoglycemia |
| GLUT | *Vm_f_*  *Km _Glcout_*  *K_eq_*  *Km _Glcin_* | 0.028 **^a^**  0.52 **^b^**  1 **^c^**  10 **^c^** | *Vm_f_*  *f_1_*  *K_eq_*  *Km _Glcout_*  *Km_Glcin_*  *f_2_*  *Km _Glcout2_*  *Km_Glcin2_* | 0.023 **^l^**  0.14 **^l^**  1 **^c^**  1.8 **^l^**  10 **^c^**  0.86 **^l^**  9.3 **^b^**  10 **^c^** | 0.028 **^l^**  0.1 **^l^**  1 **^c^**  1.8 **^l^**  10 **^c^**  0.9 **^l^**  9.3 **^b^**  10 **^c^** | 0.018 **^l^**  0.9 **^l^**  1 **^c^**  1.8 **^l^**  10 **^c^**  0.1 **^l^**  9.3 **^b^**  10 **^c^** |
| HK | *Vm_f_*  *α*  *K_m Glc_*  *Km _ATP_*  *K_eq_*  *Ki _Glc6P_*  *K_ADP_*  *Ki_Fru1,6BP_*  *α_2_* | 0.24 **^a^**  1 **^c^**  0.21 **^a^**  0.99 **^d^**  651 **^e^**  0.02 **^f^**  3.5 **^c^**  14.9 **^a^**  0.57 **^a^** | *Vm_f_*  *f1*  *K_m Glc_*  *Km _ATP_*  *K_eq_*  *Ki _Glc6P_*  *K_ADP_*  *f2*  *K_m Glc2_* | 0.036 **^l^**  0.01 **^l^**  0.03 **^l^**  1.1 **^g^**  651 **^e^**  0.02 **^f^**  3.5 **^c^**  0.99 **^l^**  0.3 **^l^** | 0.041 **^l^**  0.01 **^l^**  0.03 **^l^**  1.1 **^g^**  651 **^e^**  0.02 **^f^**  3.5 **^c^**  0.99 **^l^**  0.3 **^l^** | 0.051 **^l^**  0.6 **^l^**  0.03 **^l^**  1.1 **^g^**  651 **^e^**  0.02 **^f^**  3.5 **^c^**  0.4 **^l^**  0.3 **^l^** |
| HPI | *Vm_f_*  *Km _Glc6P_*  *Vm_r_*  *Km _Fru6P_*  *Ki_Ery4P_*  *Ki_Fru16BP_*  *Ki_6PG_*  *Ki_DHAP_* | 1.74 **^a^**  0.9 **^g^**  1.2 **^a^**  0.26 **^a^**  0.0017 **^g^**  0.17 **^g^**  0.0094 **^g^**  9.4 **^a^** | *Vm_f_*  *Km _Glc6P_*  *Vm_r_*  *Km _Fru6P_*  *Ki_Ery4P_*  *Ki_Fru16BP_*  *Ki_6PG_* | 0.24 **^l^**  0.4 **^g^**  0.54 **^l^**  0.05 **^g^**  0.001 **^g^**  0.06 **^g^**  0.015 **^g^** | 0.28 **^l^**  0.4 **^g^**  0.63 **^l^**  0.05 **^g^**  0.001 **^g^**  0.06 **^g^**  0.015 **^g^** | 0.402 **^l^**  0.4 **^g^**  0.914 **^l^**  0.05 **^g^**  0.001 **^g^**  0.06 **^g^**  0.015 **^g^** |
| PFK-I | *Vm_f_*  *Km _ATP_*  *β*  *α*  *Ka _Fru26BP_*  *Km _Fru6P_*  *L*  *Ki _CIT_*  *Ki _ATP_*  *K_ADP_*  *K_Fru16BP_*  *K_eq_* | 0.084 **^a^**  0.044 **^h^**  3.3 **^h^**  0.4 **^h^**  5.3 x10^-4^ **^h^**  5 **^h^**  18 **^h^**  5.9 **^h^**  1.75 **^h^**  5 **^c^**  5 **^c^**  247 **^i^** | *Vm_f_*  *Km _ATP_*  *β*  *α*  *Ka _Fru26BP_*  *Km _Fru6P_*  *L*  *Ki _CIT_*  *Ki _ATP_*  *K_ADP_*  *K_Fru16BP_*  *K_eq_* | 0.022 **^l^**  0.029 **^h^**  1.6 **^h^**  0.35 **^h^**  0.00085 **^h^**  0.7 **^h^**  6.6 **^h^**  10.5 **^h^**  5.8 **^h^**  5 **^c^**  5 **^c^**  247 **^i^** | 0.022 **^l^**  0.029 **^h^**  1.6 **^h^**  0.35 **^h^**  0.00085 **^h^**  0.7 **^h^**  6.6 **^h^**  10.5 **^h^**  5.8 **^h^**  5 **^c^**  5 **^c^**  247 **^i^** | 0.04 **^l^**  0.029 **^h^**  1.6 **^h^**  0.35 **^h^**  0.00085 **^h^**  0.7 **^h^**  6.6 **^h^**  10.5 **^h^**  5.8 **^h^**  5 **^c^**  5 **^c^**  247 **^i^** |
| ALDO | *Vm_f_*  *Km _Fru16BP_*  *Vm_r_*  *Km _DHAP_ Km _G3P_* | 0.12 **^a^**  0.056 **^a^**  0.09 **^a^**  0.08 **^g^**  0.16 **^g^** | *Vm_f_*  *Km _Fru16BP_*  *Vm_r_*  *Km _DHAP_*  *Km _G3P_* | 0.08 **^g^**  0.009 **^g^**  0.063 **^g^**  0.08 **^g^**  0.16 **^g^** | 0.08 **^g^**  0.009 **^g^**  0.063 **^g^**  0.08 **^g^**  0.16 **^g^** | 0.08 **^g^**  0.009 **^g^**  0.063 **^g^**  0.08 **^g^**  0.16 **^g^** |
| TPI | *Vm_f_*  *Km _DHAP_*  *Ki_Fru1,6BP_*  *α*  *Vm_r_*  *Km _G3P_* | 0.51 **^a^**  1.9 **^g^**  1.1 **^a^**  2.2 **^a^**  5.1 **^a^**  0.6 **^a^** | *Vm_f_*  *Km _DHAP_*  *Vm_f_*  *Km _G3P_* | 3.4 **^g^**  1.6 **^g^**  28 **^g^**  0.51 **^g^** | 3.4 **^g^**  1.6 **^g^**  28 **^g^**  0.51 **^g^** | 3.4 **^g^**  1.6 **^g^**  28 **^g^**  0.51 **^g^** |
| GAPDH | *Vm_f_*  *Km _NAD+_*  *Km _G3P_*  *Km _Pi_*  *Vm_r_*  *Km _1,3BPG_*  *Km _NADH_*  *Ki_Fru1,6BP_*  *α* | 3.7 **^a^**  0.08 **^g^**  0.2 **^a^**  11 **^g^**  3.3 **^a^**  0.02 **^g^**  0.004 **^g^**  12.8 **^a^**  2.3 **^a^** | *Vm_f_*  *Km _NAD+_*  *Km _G3P_*  *Km _Pi_*  *Vmr*  *Km _1,3BPG_*  *Km _NADH_* | 0.28 **^l^**  0.09 **^g^**  0.19 **^g^**  11 **^l^**  0.35 **^l^**  0.022 **^g^**  0.01 **^g^** | 0.331 **^l^**  0.09 **^g^**  0.19 **^g^**  11 **^l^**  0.413 **^l^**  0.022 **^g^**  0.01 **^g^** | 0.592 **^l^**  0.09 **^g^**  0.19 **^g^**  11 **^l^**  0.74 **^l^**  0.022 **^g^**  0.01 **^g^** |
| PGK | *Vm_f_*  *α*  *Km_1,3BPG_*  *Km _ADP_*  *Vm_r_*  *β*  *Km _3PG_*  *Km _ATP_* | 10.8 **^g^**  1 **^c^**  0.035 **^g^**  0.67 **^g^**  1.72 **^g^**  1 **^c^**  0.12 **^g^**  0.15 **^g^** | *Vm_f_*  *α*  *Km_1,3BPG_*  *Km _ADP_*  *Vm_r_*  *β*  *Km _3PG_*  *Km _ATP_* | 8.7 **^g^**  1 **^c^**  0.079 **^g^**  0.04 **^g^**  2.5 **^g^**  1 **^c^**  0.13 **^g^**  0.27 **^g^** | 8.7 **^g^**  1 **^c^**  0.079 **^g^**  0.04 **^g^**  2.5 **^g^**  1 **^c^**  0.13 **^g^**  0.27 **^g^** | 8.7 **^g^**  1 **^c^**  0.079 **^g^**  0.04 **^g^**  2.5 **^g^**  1 **^c^**  0.13 **^g^**  0.27 **^g^** |
| PGAM | *Vm_f_*  *Km _3PG_*  *Vm_r_*  *Km _2PG_* | 8 **^g^**  0.18 **^g^**  0.52 **^g^**  0.04 **^g^** | *Vm_f_*  *Km _3PG_*  *Vm_r_*  *Km _2PG_* | 0.94 **^g^**  0.19 **^g^**  0.36 **^g^**  0.12 **^g^** | 0.94 **^g^**  0.19 **^g^**  0.36 **^g^**  0.12 **^g^** | 0.94 **^g^**  0.19 **^g^**  0.36 **^g^**  0.12 **^g^** |
| ENO | *Vm_f_*  *Km _2PG_*  *Vm_r_*  *Km _PEP_* | 0.12 **^a^**  0.13 **^a^**  0.173 **^a^**  0.04 **^g^** | *Vm_f_*  *Km _2PG_*  *Vm_r_*  *Km _PEP_* | 0.34 **^g^**  0.038 **^g^**  0.38 **^g^**  0.06 **^g^** | 0.34 **^g^**  0.038 **^g^**  0.38 **^g^**  0.06 **^g^** | 0.34 **^g^**  0.038 **^g^**  0.38 **^g^**  0.06 **^g^** |
| PYK | *Vmf*  *Km _PEP_*  *Km _ADP_*  *K_eq_*  *Km _Pyr_*  *Km _ATP_* | 2.2 **^a^**  0.2 **^a^**  0.3 **^a^**  195172.4 **^j^**  10 **^k^**  0.86 **^k^** | *Vmf*  *Km _PEP_*  *Km _ADP_*  *K_eq_*  *Km _Pyr_*  *Km _ATP_* | 0.072 **^l^**  0.05 **^l^**  0.4 **^g^**  195172.4 **^j^**  10 **^k^**  0.86 **^k^** | 0.087 **^l^**  0.05 **^l^**  0.4 **^g^**  195172.4 **^j^**  10 **^k^**  0.86 **^k^** | 0.091 **^l^**  0.05 **^l^**  0.4 **^g^**  195172.4 **^j^**  10 **^k^**  0.86 **^k^** |
| LDH | *Vm_f_*  *Km _NADH_*  *Km _Pyr_*  *Vm_r_*  *Km _Lac_*  *Km _NADH_*  *α*  *β* | 0.4 **^a^**  0.00275 **^a^**  0.13 **^a^**  0.054 **^a^**  4.7 **^g^**  0.07 **^g^**  1 **^c^**  1 **^c^** | *Vm_f_*  *Km _NADH_*  *Km _Pyr_*  *Vm_r_*  *Km _Lac_*  *Km _NAD_*  *α*  *β* | 0.44 **^l^**  0.002 **^g^**  0.3 **^g^**  0.07 **^l^**  4.7 **^g^**  0.07 **^g^**  1 **^c^**  1 **^c^** | 0.468 **^l^**  0.002 **^g^**  0.3 **^g^**  0.074 **^l^**  4.7 **^g^**  0.07 **^g^**  1 **^c^**  1 **^c^** | 0.598 **^l^**  0.002 **^g^**  0.3 **^g^**  0.095 **^l^**  4.7 **^g^**  0.07 **^g^**  1 **^c^**  1 **^c^** |
| MCT1 |  |  | *Vm^f^*  *K_eq_*  *Km _Lacin_*  *Km_Lacout_* | 0.03 **^l^**  1 **^l^**  8.5 **^l^**  0.5 **^l^** | 0.03 **^l^**  1 **^l^**  8.5 **^l^**  0.5 **^l^** | 0.1 **^l^**  1 **^l^**  8.5 **^l^**  0.5 **^l^** |
| Glycogen degradation | *v*= | 1.2x10 ^-3^ **^g^** | *v*= | 4.5x10 ^-3^ **^l^** | 4.5 x10 ^-3^ **^l^** | 3x10 ^-3^ **^l^** |
| Glycogen synthesis | *v*= | 2.2x10^-3^ **^g^** | *v*= | 1x10 ^-3^ **^l^** | 1x10 ^-3^ **^l^** | 1.1x10 ^-3^ **^l^** |
| ATPases | *k*= | 3.6 x10^-3^ **^c^** | *k*= | 2.65 x 10 ^3^ **^c^** | 3.15 x 10 ^3^ **^c^** | 2.9 x 10 ^3^ **^c^** |
| AK | *k1=*  *k2=* | 1 **^c^**  2.26 **^c^** | *k1=*  *k2=* | 1 **^c^**  2.26 **^c^** | 1 **^c^**  2.26 **^c^** | 1 **^c^**  2.26 **^c^** |
| DHases | *k1=*  *k2=* | 250 **^c^**  1 **^c^** | *k1=*  *k2=* | 250 **^c^**  1 **^c^** | 250 **^c^**  1 **^c^** | 250 **^c^**  1 **^c^** |
| PPP | *v=* | 9.5x10^-5^ **^g^** | *v=* | 9.5 x 10^-5^ **^g^** | 9.5 x 10^-5^ **^g^** | 9.5 x 10^-5^ **^g^** |
| MPM | *v=* | 5 x 10^-4^ **^g^** | *v=* | 1 x 10^-4^ **^g^** | 1 x 10^-4^ **^g^** | 1 x 10^-4^ **^g^** |
| TK | *v=* | 9.5x10^-5^ **^c^** | *v=* | 9.5x 10^-5^ **^c^** | 9.5x 10^-5^ **^c^** | 9.5x 10^-5^ **^c^** |

*Km, Ka and Ki* in mM; *v*, *Vmf* and *Vmr* in µmol/min*mg of cellular protein; *k* in min*^-1^*

**^a^** Moreno-Sánchez, R., Marín-Hernández, A., Del Mazo-Monsalvo, I., Saavedra, E., Rodríguez-Enríquez, S. (2016). Assessment of the inhibitory specificity of oxamate, aminooxyacetate and dichloroacetate on energy metabolism. Submitted.

**^b^**Rodríguez-Enríquez, S., Marín-Hernández, A., Gallardo-Pérez, J.C., Moreno-Sánchez, R. (2009). Kinetics of transport and phosphorylation of glucose in cancer cells. *J Cell Physiol*. 221, 552-9.

**^c^** arbitrary or adjusted value. **These values were screened and selected when an optimum correspondence between model prediction and the experimental values (metabolites concentration and fluxes) were reached.**

**^d^** Marín-Hernández, A., Rodríguez-Enríquez, S., Vital-González, P.A, Flores-Rodríguez, F.L., Macías-Silva, M., Sosa-Garrocho, M., et al. (2006). Determining and understanding the control of glycolysis in fast-growth tumor cells. Flux control by an over-expressed but strongly product-inhibited hexokinase. *FEBS J*. 273, 1975-88.

**^e^** **The equilibrium constant at 37°C was** recalculated from the *ΔGº´* = - 16.7 KJ mol^-1^ **by using the equation *ΔGº´*= -RT ln K_eq_**.

**^f^** Wilson, J.E. (2003). Isozymes of mammalian hexokinase: structure, subcellular localization and metabolic function. *J Exp Biol*. 206, 2049-57.

**^g^** adjusted in the interval described in Marín-Hernández, A., Gallardo-Pérez, J.C., Rodríguez-Enríquez, S., Encalada, R., Moreno-Sánchez, R., Saavedra, E. (2011). Modeling cancer glycolysis. *Biochim Biophys Acta.* 1807, 755-767. doi: 10.1016/j.bbabio.2010.11.006

**^h^** Adjusted in the interval described in Moreno-Sánchez, R., Marín-Hernández, A., Gallardo-Pérez, J.C., Quezada, H., Encalada, R., Rodríguez-Enríquez, S., et al. (2012). Phosphofructokinase type 1 kinetics, isoform expression, and gene polymorphisms in cancer cells. *J Cell Biochem.* 113, 1692-703. doi: 10.1002/jcb.24039.

**^i^** **The equilibrium constant at 37°C was** recalculated from the *ΔGº´* = - 14.2 KJ mol^-1^.

**^j^** **The equilibrium constant at 37°C was** recalculated from the *ΔGº´* = - 31.4 KJ mol^-1^.

**^k^** Bergmeyer, H.U. (1983). *Methods of Enzymatic Analysis*. Winheim: Verlag Chemie.

**^l^** Marín-Hernández, A., López-Ramírez, S.Y., Del Mazo-Monsalvo, I., Gallardo-Pérez, J.C., Rodríguez-Enríquez, S., Moreno-Sánchez, R. (2014). Modeling cancer glycolysis under hypoglycemia, and the role played by the differential expression of glycolytic isoforms. *FEBS J.* 281, 3325-3345. doi: 10.1111/febs.12864
