# Supplementary material for: Inhibition of Non-flux-Controlling Enzymes Deters Cancer Glycolysis by Accumulation of Regulatory Metabolites of Controlling Steps
Source: Front Physiol. 2016 Sep 23;7:412. doi: 10.3389/fphys.2016.00412 (PMC5033973; doi:10.3389/fphys.2016.00412)
Supplement: Supplementary file 2 [file Table2.DOC]

Supplementary Material

Inhibition of non flux-controlling enzymes deters cancer glycolysis by accumulation of regulatory metabolites of controlling steps

**Álvaro Marín-Hernández*, José Salud Rodríguez-Zavala, Isis Del Mazo-Monsalvo, Sara Rodríguez-Enríquez, Rafael Moreno-Sánchez and Emma Saavedra***

*** Correspondence:** Álvaro Marín Hernández, Ph. D. and Emma Saavedra, Ph. D. e-mail: emma_saavedra2002@yahoo.com; marinhernndez@yahoo.com.mx; alvaro.marin@cardiologia.org.mx

**Supplementary Table 2. Inhibition of glycolytic enzymes by Fru1,6BP and DHAP.**

|  | Metabolites | |
| --- | --- | --- |
| Enzymes | Fru1,6BP | DHAP |
| HK | *Ki*_Fru1,6BP_ =14.9 mM  α= 0.57  Mixed-type inhibition *vs.* Glc | No effect. |
| HPI | *Ki*_Fru1,6BP_ = 60-170 µM  Competitive inhibition *vs.* Fru6P | *Ki*_DHAP_= 9.4 mM  Competitive inhibition *vs.* Fru6P |
| TPI | *Ki*_Fru1,6BP_ =1.1 mM  α= 2.2  Mixed-type inhibition *vs.* G3P |  |
| GAPDH | *Ki*_Fru1,6BP_ =12.8 mM  α= 2.3  Mixed-type inhibition *vs.* G3P |  |

Values taken from Marín-Hernández et al., 2011 and Moreno-Sánchez et al., 2016.
