# Supplementary figures and images for: Inhibition of Non-flux-Controlling Enzymes Deters Cancer Glycolysis by Accumulation of Regulatory Metabolites of Controlling Steps
Source: Front Physiol. 2016 Sep 23;7:412. doi: 10.3389/fphys.2016.00412 (PMC5033973; doi:10.3389/fphys.2016.00412)

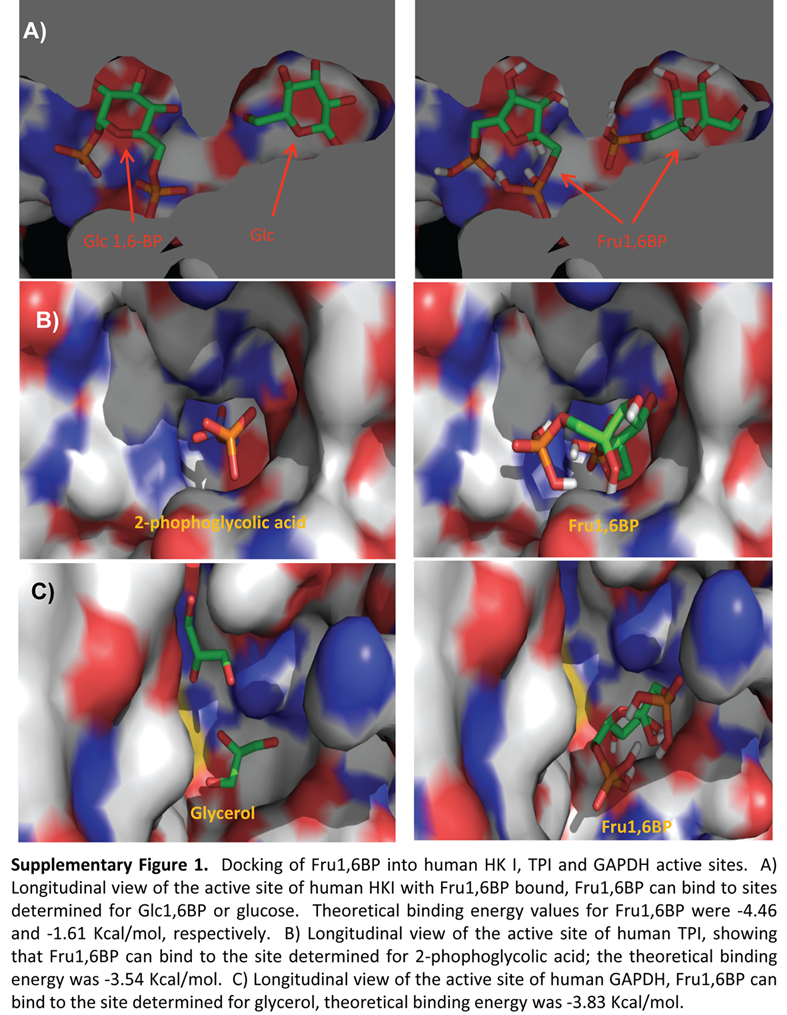

Supplement: Supplementary file 4 [file Image1.TIF]
